# Targeting Epstein-Barr virus oncoprotein LMP1-mediated high oxidative stress suppresses EBV lytic reactivation and sensitizes tumors to radiation therapy

**DOI:** 10.7150/thno.46006

**Published:** 2020-10-25

**Authors:** Jianmin Hu, Yueshuo Li, Hongde Li, Feng Shi, Longlong Xie, Lin Zhao, Min Tang, Xiangjian Luo, Weihua Jia, Jia Fan, Jian Zhou, Qiang Gao, Shuangjian Qiu, Weizhong Wu, Xin Zhang, Weihua Liao, Ann M. Bode, Ya Cao

**Affiliations:** 1 Key Laboratory of Cancer Carcinogenesis and Invasion, Chinese Ministry of Education, Xiangya Hospital, Central South University, Changsha 410078, China.; 2 Cancer Research Institute and School of Basic Medicine Science, Xiangya School of Medicine, Central South University, Changsha 410078, China.; 3 Key Laboratory of Carcinogenesis, Chinese Ministry of Health, Changsha 410078, China.; 4 Molecular Imaging Research Center of Central South University, Changsha 410078, China.; 5 Research Center for Technologies of Nucleic Acid-Based Diagnostics and Therapeutics, Changsha 410078, China.; 6 National Joint Engineering Research Center for Genetic Diagnostics of Infectious Diseases and Cancer, Changsha 410078, China.; 7 State Key Laboratory of Oncology in South China, Collaborative Innovation Center for Cancer Medicine, Guangdong Key Laboratory of Nasopharyngeal Carcinoma Diagnosis and Therapy, Sun Yat-sen University Cancer Center, Guangzhou, 510060, China.; 8 Key Laboratory of Cancer Carcinogenesis and Invasion, Chinese Ministry of Education, Zhongshan Hospital, Shanghai Medical School, Fudan University, Shanghai 200000, China.; 9 Department of Otolaryngology Head and Neck Surgery, Xiangya Hospital, Central South University, Changsha 410078, China.; 10 Department of Radiology, Xiangya Hospital, Central South University, Changsha 410078, China.; 11 The Hormel Institute, University of Minnesota, Austin, MN 55912, USA.

**Keywords:** oxidative stress, Epstein-Barr virus reactivation, radioresistance, prognostic bio-model, nasopharyngeal carcinoma

## Abstract

Generating oxidative stress is a critical mechanism by which host cells defend against infection by pathogenic microorganisms. Radiation resistance is a critical problem in radiotherapy against cancer. Epstein-Barr virus (EBV) is a cancer-causing virus and its reactivation plays an important role in the development of EBV-related tumors. This study aimed to explore the inner relationship and regulatory mechanism among oxidative stress, EBV reactivation, and radioresistance and to identify new molecular subtyping models and treatment strategies to improve the therapeutic effects of radiotherapy.

**Methods:** ROS, NADP^+^/NADPH, and GSSG/GSH were detected to evaluate the oxidative stress of cells. 8-OHdG is a reliable oxidative stress marker to evaluate the oxidative stress in patients. Its concentration in serum was detected using an ELISA method and in biopsies was detected using IHC. qPCR array was performed to evaluate the expression of essential oxidative stress genes. qPCR, Western blot, and IHC were used to measure the level of EBV reactivation *in vitro* and *in vivo*. A Rta-IgG ELISA kit and EBV DNA detection kit were used to analyze the reactivation of EBV in serum from NPC patients. NPC tumor tissue microarrays was used to investigate the prognostic role of oxidative stress and EBV reactivation. Radiation resistance was evaluated by a colony formation assay. Xenografts were treated with NAC, radiation, or a combination of NAC and radiation. EBV DNA load of tumor tissue was evaluated using an EBV DNA detection kit. Oxidative stress, EBV reactivation, and the apoptosis rate in tumor tissues were detected by using 8-OHdG, EAD, and TUNEL assays, respectively.

**Results:** We found that EBV can induce high oxidative stress, which promotes its reactivation and thus leads to radioresistance. Basically, EBV caused NPC cells to undergo a process of 'Redox Resetting' to acquire a new redox status with higher levels of ROS accumulation and stronger antioxidant systems by increasing the expression of the ROS-producing enzyme, NOX2, and the cellular master antioxidant regulator, Nrf2. Also, EBV encoded driving protein LMP1 promotes EBV reactivation through production of ROS. Furthermore, high oxidative stress and EBV reactivation were positively associated with poor overall survival of patients following radiation therapy and were significant related to NPC patients' recurrence and clinical stage. By decreasing oxidative stress using an FDA approved antioxidant drug, NAC, sensitivity of tumors to radiation was increased. Additionally, 8-OHdG and EBV DNA could be dual prognostic markers for NPC patients.

**Conclusions:** Oxidative stress mediates EBV reactivation and leads to radioresistance. Targeting oxidative stress can provide therapeutic benefits to cancer patients with radiation resistance. Clinically, we, for the first time, generated a molecular subtyping model for NPC relying on 8-OHdG and EBV DNA level. These dual markers could identify patients who are at a high risk of poor outcomes but who might benefit from the sequential therapy of reactive oxygen blockade followed by radiation therapy, which provides novel perspectives for the precise treatment of NPC.

## Introduction

Reactive oxygen species (ROS)-mediated oxidative stress has an increasingly well-recognized broad function in oncogenesis 1. Generating oxidative stress is a critical mechanism by which host cells defend against infection by pathogenic microorganisms 2. Several reports have shown that both DNA and RNA viruses can lead to oxidative stress by encoding various products and play roles in virus survival. For example, HBV encoded HBx 3 and HSV-1 encoded UL12.5 4 can induce oxidative stress by degradation of mtDNA; KSHV encoded Rac-1 5 and HIV encoded Gp120, Tet or Nef 6 can induce oxidative stress by regulating the NOX pathway.

EBV, a member of γ-herpes viruses, is an important cancer-causing virus 7-9. Cancer associated with EBV account for approximately 1.5% of all cancers, and represent 1.8% of all cancer deaths worldwide 10. It is reported that several EBV-encoded products, including EBNA1, EBER and LMP1, can also induce oxidative stress 11-13. The mechanism of inducing oxidative stress by EBNA1 was via activating of NOX pathway, while the detailed mechanism by LMP1 remains to be studied.

Like all herpesviruses, EBV establishes a latent infection that is periodically reactivated into the productive lytic cycle 14. The latent-lytic switch is tightly controlled by both cellular and viral factors. In recent years, evidence of a contribution of the lytic cycle to EBV-induced oncogenesis has emerged and has become a hot topic in this field 15-17. The products that are encoded by EBV in the lytic reactivation mainly contribute to the induction of genome instability 18-20, the counteraction of host immune responses 21,22, the resistance to cell death 17,23,24, and the promotion of tumor development, progression, and invasiveness 25. Therefore, the regulation mechanism of EBV reactivation and whether the above-mentioned oxidative stress plays roles in EBV reactivation remains to be fully investigated.

NPC is an infection-associated cancer strongly driven by EBV 8,15,26,27. According to the International Agency for Research on Cancer, there were about 129000 new cases of nasopharyngeal carcinoma, accounting for 0.7% of all cancers diagnosed in 2018^10^. Although the widespread application of intensity modulated radiotherapy and optimization of chemotherapy strategies (induction, concurrent, adjuvant) have contributed to improved survival with reduced toxicities, radiation resistance has been a major hurdle that limits therapeutic efficacy and results in recurrence 26,27. Several groups have studied the factors that lead to resistance of NPC to radiation, such as metabolic reprogramming 28-30, DNA damage and repair 31, and angiogenesis 32. Additionally, whether oxidative stress-induced EBV reactivation plays a role in NPC radioresistance is poorly understood.

In this study, we aimed to (i) determine the oxidative stress status in NPC patients and NPC cells including EBV latent infection and primary infection, (ii) examine the mechanisms of high oxidative stress in EBV positive cells, (iii) delineate the relationship between oxidative stress and EBV reactivation, (iv) analyze the effect of LMP1 on ROS production and EBV lytic reactivation, (v) explore whether high oxidative stress and EBV reactivation can lead to poor prognosis, and (vi) elucidate the prospect of clinical application including the *in vivo* antitumor effect and the possibility to build new molecular subtyping models.

## Methods

### Cell lines and culture conditions

The human NPC cell lines, HK1, HK1-EBV, HONE1, HONE1-EBV, CNE1, CNE1-LMP1, C666-1, C666-1shLMP1 which have been described previously 33-35 can reflect the genuine type Ⅱ latency of NPC. The NPC cell lines were cultured in RPMI-1640 medium (Cat: 11875500, Hyclone, Logan, UT, USA) supplemented with 10% fetal bovine serum (Cat: 04-001-1, BI, Kibbutz Beit-Haemek, Israel), penicillin (100 U) and streptomycin (100 mg/mL) in a 37 °C incubator with a humidified, 5% CO2 atmosphere. In addition, in order to maintain the stability of the EBV genome, 500 µg/mL G418 (Cat: 11811031, Thermo Fisher Scientific, Waltham, MA, USA) were added to the medium of the HK1-EBV and HONE1-EBV cells.

### Clinical specimens

The serum samples of NPC patients were obtained from School of Public Health & Cancer Center, Sun Yat-Sen University, Guangzhou, China, and the clinical-pathological information for these patients is summarized in Table S1. Nasopharyngitis (NP) and NPC tissues were collected from the Department of Pathology at Xiangya Hospital, Central South University, Changsha, China, and the clinical-pathological information for these patients is summarized in Table S2. The study protocol was approved by the ethical review committees of the two hospitals. All clinical data were obtained from the hospital pathologic records. Among them, tumor differentiation was characterized according to WHO classification, whereas the pathologic stage was analyzed according to the TNM classification system of the International Union against Cancer. Paraffin-embedded NPC tissue microarrays (containing 92 cases of NPC and 30 cases of NP patients) were purchased from Pantomics (NPC 1501 and NPC 1502, Richmond, CA) and follow-up data and clinical details are shown in Table S3. Another NPC tissue microarray (containing 129 cases of NPC) was purchased from Shanghai Outdo Biotech Co. (HNasN129Su01, Shanghai, China) and follow-up data and clinical details are shown in Table S4.

### Drug treatment

Due to the low level of spontaneous lytic reactivation of EBV was observed in HK1-EBV and HONE1-EBV positive cell lines, the cells were treated with TPA/NaB (40 ng/mL TPA and 6 mM NaB for HK1-EBV cells and 20 ng/mL TPA and 3 mM NaB for HONE1-EBV cells) for 48 h to induce EBV lytic reactivation or with an equivalent of DMSO as negative control. To inhibit ROS generation, cells were treated with 5 mM N-acetyl cysteine (NAC) for 1 h before treatment with TPA/NaB. To induce ROS generation, cells were treated with 50 μM H_2_O_2_ for 48 h. TPA, NaB, NAC and H_2_O_2_ were all brought from Sigma (Sigma-Aldrich, St. Louis, MO, USA).

### Plasmids and siRNA

The *NOX2* siRNAs were designed and synthesized by RIBOBIO (Gaungzhou, China) and their specific sequences were listed in Table S5. The *NOX2* knockdown efficiency was verified in Figure S2. The pSG5-based expression vector for LMP1 was kindly provided by Dr. Kenneth M Izumi (Brigham and Women's Hospital, Boston, MA, USA). The *LMP1* siRNAs were designed and synthesized by TSING KE (Beijing, China) according to the sequence of EBV origin Akata-EBV cells and their specific sequences were listed in Table S5. Plasmid transfections were performed using Lipofectamine 3000 (Invitrogen, Carlsbad, CA, USA) from Invitrogen according to the manufacturer's protocol. The *LMP1* overexpression and knockdown efficiency were verified in Figure S3.

### ROS level measurements

Total intracellular ROS levels were assessed by using a CellROX Deep Red reagent for oxidative stress detection (Cat: C10422, Thermo Fisher Scientific, Waltham, MA, USA) according to the manufacturer's instructions. Briefly, the cells after treatment were resuspended in PBS. The CellROX Deep Red reagent was added at a final concentration of 5 μM, and the mixture was incubated for 60 min at 37 °C in 5% CO_2_ while protected from light. After they were washed three times in PBS, the cells were collected, and the CellROX Deep Red signal was detected using flow cytometry.

### NADP^+^/NADPH measurements

Intracellular NADP^+^/NADPH levels were assayed using an NADP^+^/NADPH assay kit (Cat: K347-100, Biovision**,** Zurich, Switzerland) according to the manufacturer's instructions. The total protein concentrations of samples run in parallel were measured using a BCA protein assay kit (BBI, China). The total NADPH concentration was normalized to protein content.

### GSSG/GSH measurements

Total glutathione (GSH) and oxidized glutathione (GSSG) were measured using a GSH/GSSG assay kit (Cat: EGTT-100, BioAssay Systems, CA, USA) according to the manufacturer's protocol. The total protein concentrations of samples run in parallel were measured using a BCA protein assay kit (BBI, China). The total glutathione concentration was normalized to protein content.

### ELISA analysis of 8-OHdG and Rta-IgG concentrations

Serum samples obtained from NPC patients and healthy control subjects were collected using serum separator tubes and centrifuged at 3,000 g for 10 min within 4 h. The supernatant fraction was stored at -80 °C before the final analysis. The levels of 8-hydroxydeoxyguanosine (8-OHdG), an important and reliable marker of oxidative stress, were measured using an ELISA kit (Cat: STA-320, Cell Biolabs Inc, San Diego, CA, USA) and conducted according to the manufacturer's guidelines. The kit has an 8-OHdG detection sensitivity range of 100 pg/mL to 20 ng/mL. The levels of Rta-IgG were measured using an ELISA kit (Cat: A-0496, Tarcine, Beijing, China) and conducted according to the manufacturer's guidelines.

### IHC analysis of an NPC tissue array and NPC biopsies

Immunohistochemistry (IHC) was performed as previously described 36. Antibodies included anti-LMP1 (diluted 1:50, #ab78113, Abcam, Cambridge, MA, USA), anti-8-OHdG (diluted 1:3000, #ab48508, Abcam, Cambridge, MA, USA), anti-EAD (diluted 1:100, #MAB8186, Merck Millipore, Darmstadt, Germany) and anti-BHRF1 (diluted 1:200, #MAB8188, Merck Millipore). Images of the sections were acquired and scores of each section were quantified by two pathologists in the Xiangya Hospital (Changsha, China). The cells with 8-OHdG nuclear signal while LMP1 and BHRF1 cytoplasmic signal were calculated as positive cells.

### EBV infection assay

The EBV infection assay was performed as previously described 33,37. Briefly, upon lytic reactivation of EBV in HK1-EBV and HONE1-EBV cells by TPA/NaB treatment for 48 h, cells were centrifuged at 115 × g for 5 min and the culture supernatants were filtered with 0.45 μm filter membrane (Millipore). Then, the supernatant was centrifuged at 37,500 × g for 4 h at 4 °C to concentrate the viral particles. The supernatant was discarded, and the pellet was resuspended with fresh RPMI-1640 medium in 1/10 of its original volume. The medium containing viral particles was then used to infect EBV negative HK1 cells (48 h and 120 h) and HONE1 cells (48 h and 96 h). The infected cells were harvested at different time points and subjected to RNA extraction to determine EBV gene expression by real-time PCR, and subjected to flow cytometry to detect total ROS level as described before.

### RNA isolation and real-time PCR

Total RNA was isolated from cultured cells with the NucleoZOL total RNA extraction reagent (Cat: 740404, MACHEREY-NAGEL GmbH & Co. KG, Düren, Germany) according to the manufacturer's instructions. The RevertAid First Strand cDNA Synthesis Kit (Cat: K1622, Invitrogen, Carlsbad, CA, USA) was used for reverse transcription. Quantitative real-time PCR was performed in triplicate using FastStart Universal SYBR Green Master Mix (Cat: A25742, Invitrogen) on Real-time System (ABI7500, Applied Biosystems, USA). The primers are list in Table S6 and relative gene expression was calculated using the 2^-ΔΔct^ method after normalization to the reference gene *β-actin*.

### Western blot analysis and antibodies

Cells were harvested and disrupted in IP lysis buffer (25 mM Tris-HCl, pH 7.4, 150 mM NaCl, 1% NP40, 1 mM EDTA, 5% glycerol). Extracted proteins were separated by SDS-PAGE and transferred onto PVDF membranes. Binding of primary antibodies was detected using peroxidase-conjugated secondary antibodies. Visualization was performed using the ChemiDoc XRS system with Image Lab software (Bio-Rad, CA, USA). Antibodies included anti-LMP1 (diluted 1:1000, #ab78113, Abcam, Cambridge, MA, USA), anti-Zta (diluted 1:200, #sc-53904, Santa Cruz Biotechnology, Dallas, TX, USA), anti-EAD (diluted 1:3000, #MAB8186, Merck Millipore, Darmstadt, Germany), anti-NOX2 (diluted 1:2000, #19013-1-AP, Proteintec, Rosemont, USA), anti-Nrf2 (diluted 1:1000, #ab137550, Abcam, Cambridge, MA, USA) and anti-β-actin (diluted 1:3000, Santa Cruz Biotechnology, Dallas, TX, USA).

### EBV copy number quantification

The virion-derived DNA was measured to reflect reactivation. Briefly, cell supernatant was harvested and free DNA in the medium was removed by DNase I (room temperature, 15 min) and then the DNase I (65 °C, 15 min) was inactivated. Next, the cell supernatant DNA was extracted using the QIAamp DNA Mini Kit (Cat: 51306, QIAGEN, Chatsworth, CA) according to the kit handbook.

To quantify EBV copy numbers, the given standard samples were first used to create the standard curve according to the manufacturer's instructions by using an EBV fluorescence quantitative PCR diagnostic kit (Shengxiang, Changsha, China). Then 10 μL of each DNA sample were added to PCR reaction tubes, and PCR was performed as follows (ABI7500, Applied Biosystems, USA): 50 °C for 2 min; 94 °C for 5 min; 94 °C for 15 s and 57 °C for 31 s (data collection) for 45 cycles; 25 °C for 10 s. The EBV copy number of each sample can be calculated by the corresponding threshold (CT) cycle with the aid of the standard curve.

### Colony formation and radiosensitivity assays

NPC cells were seeded in 6-well plates in triplicate and treated as indicated and exposed to different doses of irradiation. After 10-14 days, cells were washed with 1 × PBS, fixed in methanol for 20 min and stained with crystal violet for 15 min at room temperature. Colonies containing more than 50 cells were counted using Image J software and the survival fractions were calculated. The survival curves were drawn using GraphPad Prism 5 software (GraphPad Software, La Jolla, CA, USA).

### Tumorigenicity assay

All animal experiments were approved by the Medical Ethics Committee of Xiangya Hospital, Central South University, following the Guidelines of Animal Handling and Care in Medical Research from Hunan Province, China. Briefly, HONE1-EBV cells (5×10^6^) were injected into the subcutaneous tissue over the right flank region of nude mice (BCLB/c-nu, female, 4 weeks old). Tumors grew to an average volume of 120 mm^3^ prior to initiation of therapy. HONE1-EBV xenograft bearing mice were randomly assigned into 4 groups (n = 5) as follows: (1) vehicle control (0.9% saline buffer, 100 μL, i.p., every other day for 3 weeks); (2) NAC (150 mg/kg, 100 μL, i.p., every other day for 3 weeks); (3) radiation (2 Gy, twice per week for 2 weeks) was delivered to the tumor; (4) NAC (150 mg/kg, 100 μL, i.p., every other day for 3 weeks) plus ionizing radiation (2 Gy, twice per week for two weeks, 3 h after NAC injection). Tumor volume was assessed by caliper measurements every other day and calculated according to the formula: V= L × W^2^/2 (L: tumor length, W: tumor width). At the end of experiments, mice were euthanized and the tumor weights were recorded. The expression levels of Ki67, 8-OHdG, EAD and BHRF1 were detected by IHC as described previously 36. In addition, the paraffin-embedded tumor tissue sections were stained with a terminal deoxynucleotidyl transferase-mediated dUTP nick-end labeling (TUNEL) assay kit (Yesen, Shanghai, China) according to the manufacturer's instructions to detect apoptosis of tumor cells.

### Statistical analysis

All statistical analyses were performed using SPSS 18.0 statistical software (SPSS Inc., Chicago, IL, USA). We statistically evaluated the experimental results by using the Student's t test, the Pearson's χ^2^ test, ANOVA, and Kaplan-Meier Survival analysis. The data were expressed as mean ± standard deviation (S.D.). A value of *p* < 0.05 was considered statistically significant.

## Results

### EBV induces high oxidative stress in NPC patients and cells

**Figure 1:**
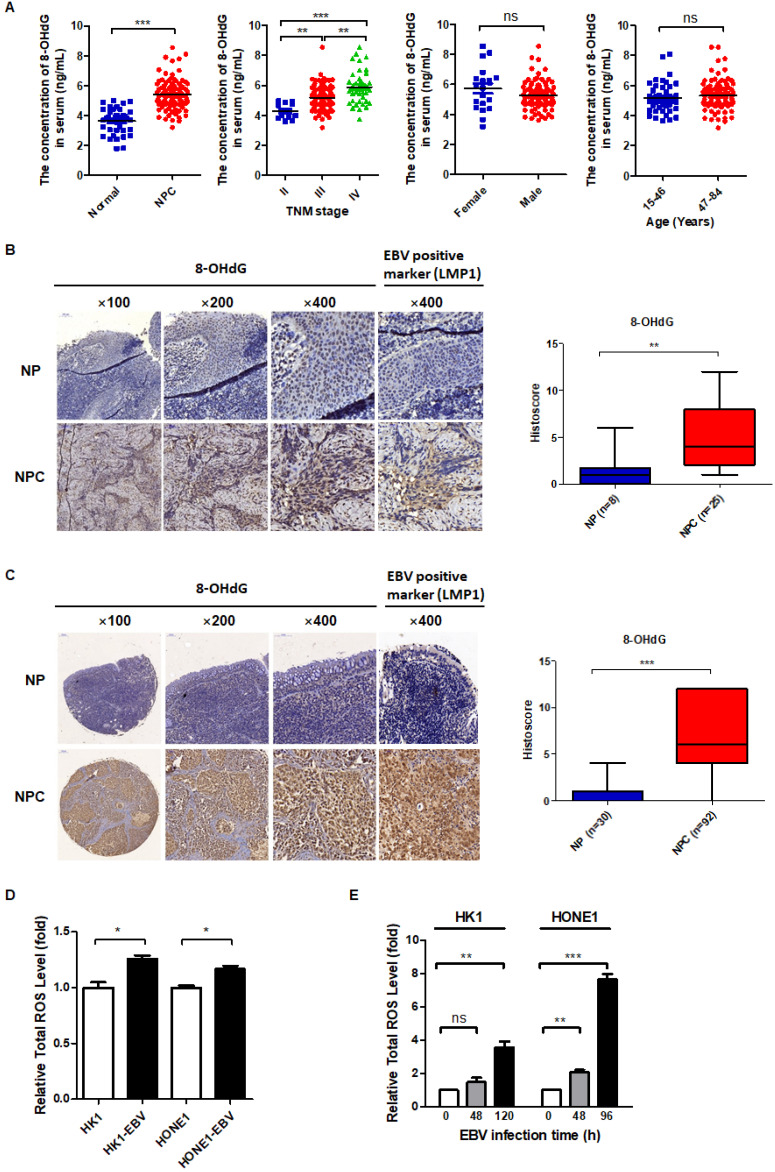
** EBV induces high oxidative stress in NPC patients and cells. (A)** Serum 8-OHdG concentration of NPC patients (n = 95) and healthy subjects (n = 40) was assessed by the ELISA method (**p < 0.01, ***p < 0.001; ns: no significant difference). **(B)** (*Left*) Representative IHC staining of 8-OHdG expression from tissue slices of 25 NPC patients and 8 NP patients. NP: nasopharyngitis; NPC: nasopharyngeal carcinoma. (*Right*) 8-OHdG level is calculated in NPC and NP patients (**p < 0.01). **(C)** (*Left*) Representative IHC staining of 8-OHdG expression from a tissue microarray of 92 NPC patients and 30 NP patients. (*Right*) 8-OHdG level is calculated in NPC and NP patients (***p < 0.001). **(D)** ROS levels of EBV-positive and -negative cells were detected by FCM using CellROX Deep Red. Data are shown as means ± S.D.; n = 3; *p < 0.05.** (E)** EBV negative cells were infected by EBV as the methods described in Materials and methods. The medium containing viral particles was then used to infect EBV negative HK1 cells (48 h and 120 h) and HONE1 cells (48 h and 96 h). The ROS levels of cells before infection and after infection at different time points were detected by FCM using CellROX Deep Red. Data are shown as means ± S.D.; n = 3; ns: no significant difference. **p < 0.01, ***p < 0.001.

Cancer cells are characterized by high levels of oxidative stress 38. This oxidative stress is exerted by reactive oxygen species (ROS) that accumulate as a result of an imbalance between ROS generation and elimination 39. In order to determine whether EBV plays a causative role in inducing oxidative stress, we first detected the oxidative stress level in NPC patients compared with NP patients. 8-hydroxydeoxyguanosine (8-OHdG) is an important and reliable marker of oxidative stress and can be used to estimate the level of oxidative stress in clinical samples 40. We collected the serum from 95 patients with a primary diagnosis of NPC and 40 healthy control subjects, and then detected the concentration of 8-OHdG by an ELISA method. The results showed that 8-OHdG concentration was much higher in NPC patients compared to healthy control subjects and was positively correlated with TNM stage, but had no correlation with patients' age and sex (Figure 1A). Then we collected tissue slices from 25 NPC patients and 8 NP patients and examined the level of 8-OHdG by IHC. The results showed that the level of oxidative stress was much higher in NPC patients compared to NP patients (Figure 1B). Additionally, an NPC tissue microarray, consisting of samples from 92 NPC patients and 30 NP patients, was used to further confirm the conclusion. The level of 8-OHdG was detected by IHC and the results showed the level of oxidative stress was indeed much higher in NPC patients compared to NP patients (Figure 1C).

To determine whether EBV promotes ROS production, the total ROS level was compared between EBV positive cells and the negative cells. The results showed that EBV-positive cells have higher total cellular ROS compared to EBV-negative cells (Figure 1D). In addition, we also detected the ratios of the main redox pairs of NADP^+^/NADPH and GSSG/GSH, which are known to reflect the level of oxidative stress. We found that EBV clearly increased the NADP^+^/NADPH and GSSG/GSH ratios (Figure S1A-B). Further, we also conducted EBV primary infection to determine whether EBV infection induces oxidative stress. The infection efficiency was verified by determination of EBV genes in EBV infected cells at different time points (Figure S1C-D). As shown in Figure 1E, EBV primary infection also induced oxidative stress.

Overall, both *in vitro,* including latent infection and primary infection and *in vivo* data, including serum and tissue biopsies of patients demonstrated that EBV induces high oxidative stress.

### EBV upregulates the expression of cellular NOX2 and Nrf2

**Figure 2:**
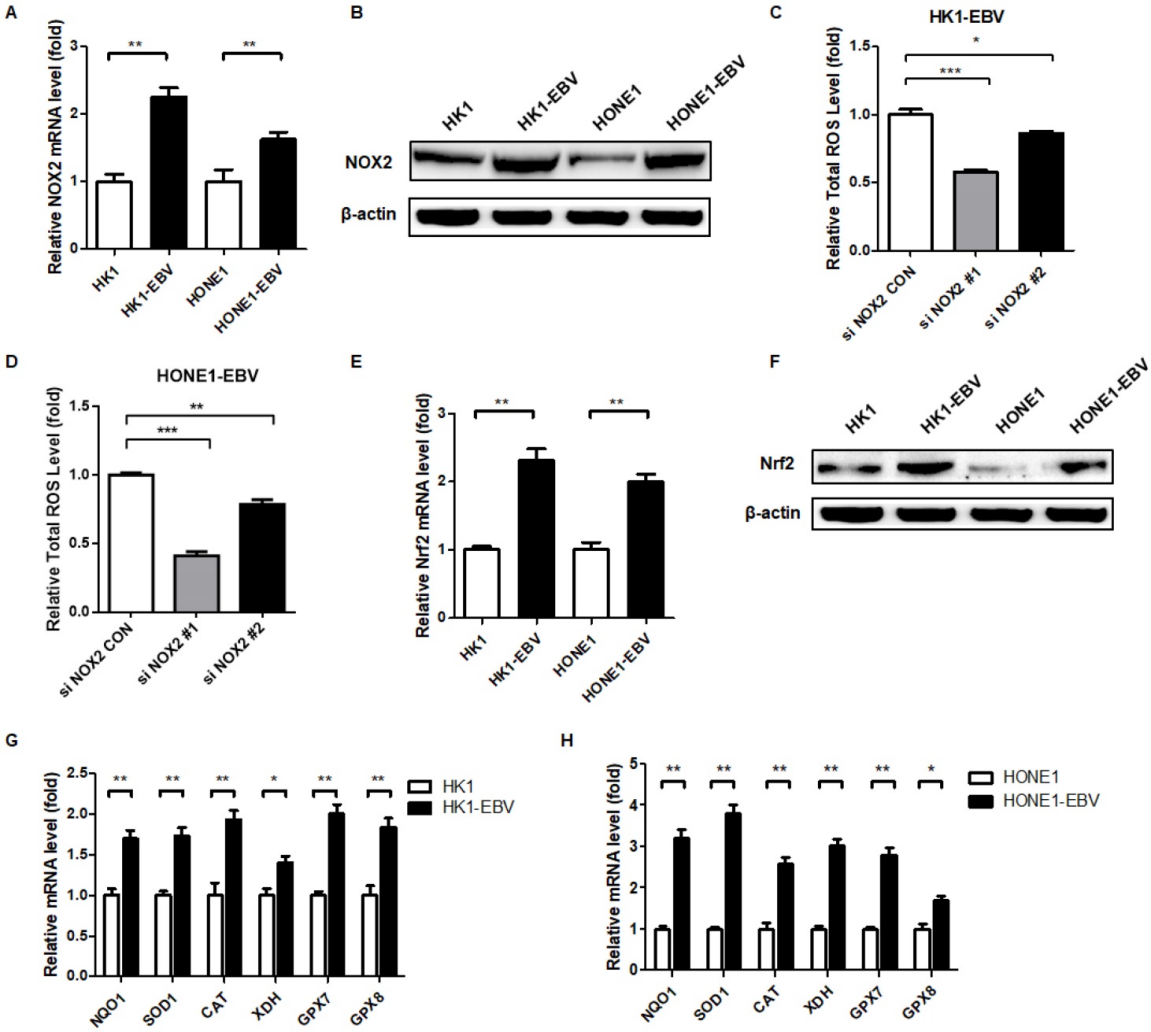
** EBV upregulates the expression of NOX2 and Nrf2. (A)** The mRNA levels of *NOX2* in EBV-positive and -negative cells were detected by RT-PCR. Data are shown as means ± S.D.; n = 3; **p < 0.01. **(B)** NOX2 protein expression was detected by western blot analysis and β-actin was used as an internal control. **(C-D)** The NOX2 of EBV-positive cells were treatment with siNOX2, and ROS levels were detected by FCM using CellROX Red regent. Data are shown as means ± S.D.; n = 3; *p < 0.05, **p < 0.01, ***p < 0.001. **(E)** The mRNA levels of *Nrf2* in EBV-positive and -negative cells were detected by RT-PCR. Data are shown as means ± S.D.; n = 3; **p < 0.01. **(F)** Nrf2 protein expression was detected by western blot analysis and β-actin was used as an internal control. **(G-H)** The transcription levels of Nrf2 target genes *NQO1, SOD1, CAT, XDH, GPX7*, and *GPX8* were detected by RT-PCR. Data are shown as means ± S.D.; n = 3; *p < 0.05, **p < 0.01.

To explore the cause of high oxidative stress in EBV-positive cells, we measured ROS-producing and -eliminating factors. The family of NADPH oxidases, now collectively known as NOX enzymes, is an important intracellular source of ROS generation 41,42. We detected the expression of NOX enzymes in EBV-positive and -negative cells and found that NOX2 was significantly elevated in EBV-positive cells compared to EBV-negative cells (Figure 2A-B). To further characterize the effect of NOX2 on intracellular ROS levels, we used siRNA targeting NOX2 in HK1-EBV and HONE1-EBV cells. Using flow cytometry, we revealed that EBV-induced ROS generation was inhibited by siNOX2 systems (Figure 2C-D). These observations suggested that EBV-induced ROS elevation was at least partly caused by activation of NOX2.

To combat the elevated ROS, the cell is equipped with various potent antioxidant defenses. The concomitant upregulation of antioxidant pathways prevents ROS-mediated cytotoxicity and can enhance tumor survival 39,43. Nuclear factor erythroid 2-related-factor 2 (Nrf2) is a master transcriptional regulator of antioxidant responses 39. Therefore, we detected the expression and activity of Nrf2 in EBV-negative and -positive cells. EBV-positive cells exhibited higher levels of Nrf2 compared to EBV-negative cells (Figure 2E-F). Also, Nrf2 target antioxidant genes were also elevated in EBV-positive cells, which reflects the response of antioxidant pathways to combat the cytotoxicity of ROS to cells (Figure 2G-H). Therefore, EBV appears to lead the NPC cells to undergo a process of “Redox Resetting” to acquire a new redox status to tolerate higher levels of ROS accumulation and stronger antioxidant systems by increasing the expression of the ROS-producing enzyme, NOX2, and the cellular master antioxidant regulator, Nrf2.

### EBV-induced high oxidative stress promotes its reactivation

**Figure 3:**
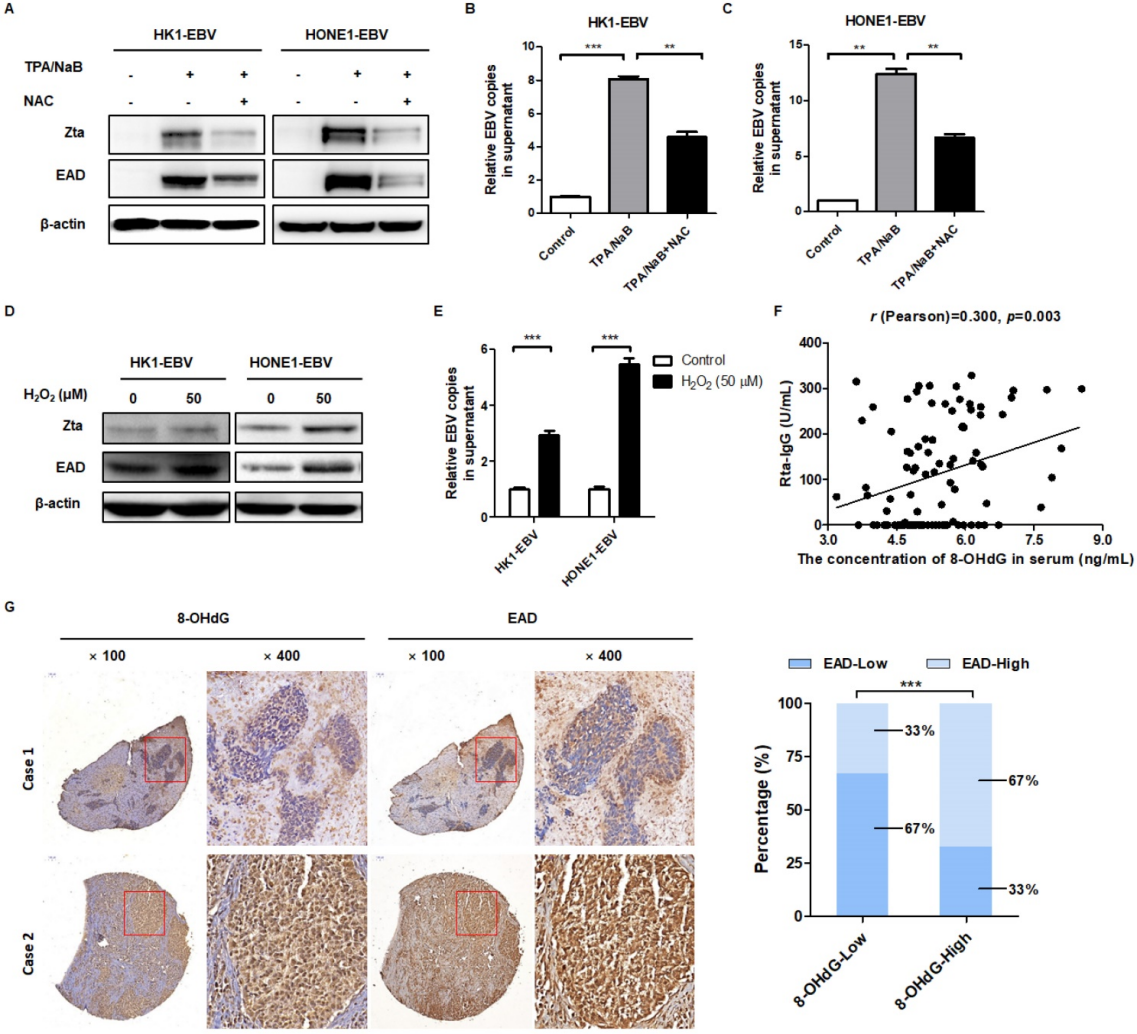
** EBV-induced high oxidative stress promotes its reactivation. (A)** Cells were treated with NAC (5 mM) for 1 h and followed by TPA/NaB induction. The EBV lytic marker Zta and EAD protein expression were detected by western blot and β-actin was used as an internal control. **(B-C)** The viral genomes of EBV positive cells after treatment were detected by using EBV fluorescence quantitative PCR diagnostic kit. Data are shown as means ± S.D.; n = 3; **p < 0.01, ***p < 0.001. **(D-E)** Cells were treated with H_2_O_2_ (50 µM) for 24 h, the protein expression and viral genomes of EBV-positive cells after treatment were detected using the same methods as described before. Data are shown as means ± S.D.; n = 3; ***p < 0.001. **(F)** Serum Rta-IgG and 8-OHdG concentrations in the serum of NPC patients (n = 95) were assessed using the ELISA method. The correlation between 8-OHdG and Rta-IgG is shown by scatter plot; *r* (Pearson) = 0.300, *p* = 0.003. **(G)** *(left)* Representative IHC photographs showing the expression of 8-OHdG and EAD in consecutive sections of NPC microarrays (NPC 1501 and NPC 1502, Richmond, CA). *(right)* EAD level is calculated based on 8-OHdG expression in NPC microarrays (***p < 0.001).

In order to determine whether oxidative stress is involved in EBV reactivation, cells were treated with the antioxidant NAC before induction of EBV reactivation. To examine the effect of EBV lytic cycle induction in NPC cells, a panel of EBV-positive NPC cell lines was assayed for expression of EBV lytic proteins (Zta and EAD) and replication of EBV DNA in cell supernatant by western blot and qPCR, respectively. As expected, TPA/NaB significantly induced Zta and EAD expression (Figure 3A). Also, the relative EBV copies in cell supernatant were also increased which demonstrated the EBV lytic reactivation were induced effectively (Figure 3B-C). While addition of NAC significantly reduced the level of TPA/NaB-induced EBV lytic reactivation (Figure 3A-C, the third panel). In addition, cells were treated with ROS inducer H_2_O_2_. After treatment, EBV lytic reactivation were induced effectively as evidenced by the expression of EBV lytic proteins and relative EBV copies in cell supernatant (Figure 3D-E). These evidences showed that ROS is indeed involved in the regulation of EBV reactivation.

Next, we further explored the inner relationship between oxidative stress and EBV reactivation in NPC patients. We detected Rta-IgG, which was positively correlated with EBV reactivation, and the content of 8-OHdG in the serum of 95 patients with NPC primary diagnosis, to analyze their relationship. The *r* (Pearson) is 0.300, which revealed that 8-OHdG was positively correlated with Rta-IgG in human NPC patients (Figure 3F). Furthermore, two NPC tissue microarrays were used to exam the correlation between oxidative stress marker 8-OHdG and EBV lytic reactivation marker EAD. The results showed that 8-OHdG expression was positively correlated with EAD in human NPC patients (Figure 3G and Figure S3A-B, Table 1 and Table S7).

These data supported the contention that ROS are involved in EBV reactivation and the level of oxidative stress is positively correlated with EBV reactivation both in serum and biopsies of NPC patients.

### LMP1 promotes EBV reactivation through production of ROS

EBV-encoded *LMP1* is a driving oncogene in NPC 16. First, the role of LMP1 on ROS was conducted. The LMP1 overexpression and knockdown strategy was performed and their efficiency was verified in Figure S4A-E. As shown in Figure S4F-G, overexpression of LMP1 significantly increased the level of ROS. CNE1-LMP1 is an NPC cell line which was stably overexpressed LMP1. We also found that CNE1-LMP1 has a higher level of ROS than CNE1 (Figure S4H). Then we transfected cells with LMP1 siRNA pool or negative siRNA and determined the role of LMP1 on ROS generation. The results showed that LMP1 depletion significant decrease the level of ROS (Figure S4I-J). C666-1 shLMP1 is an NPC cell line which LMP1 was stable knockdown. We found that the C666-1 shLMP1 has a lower level of ROS than C666-1 (Figure S4K). The above evidence showed that LMP1 promotes ROS generation both in transient transfection and stable cell lines.

**Figure 4:**
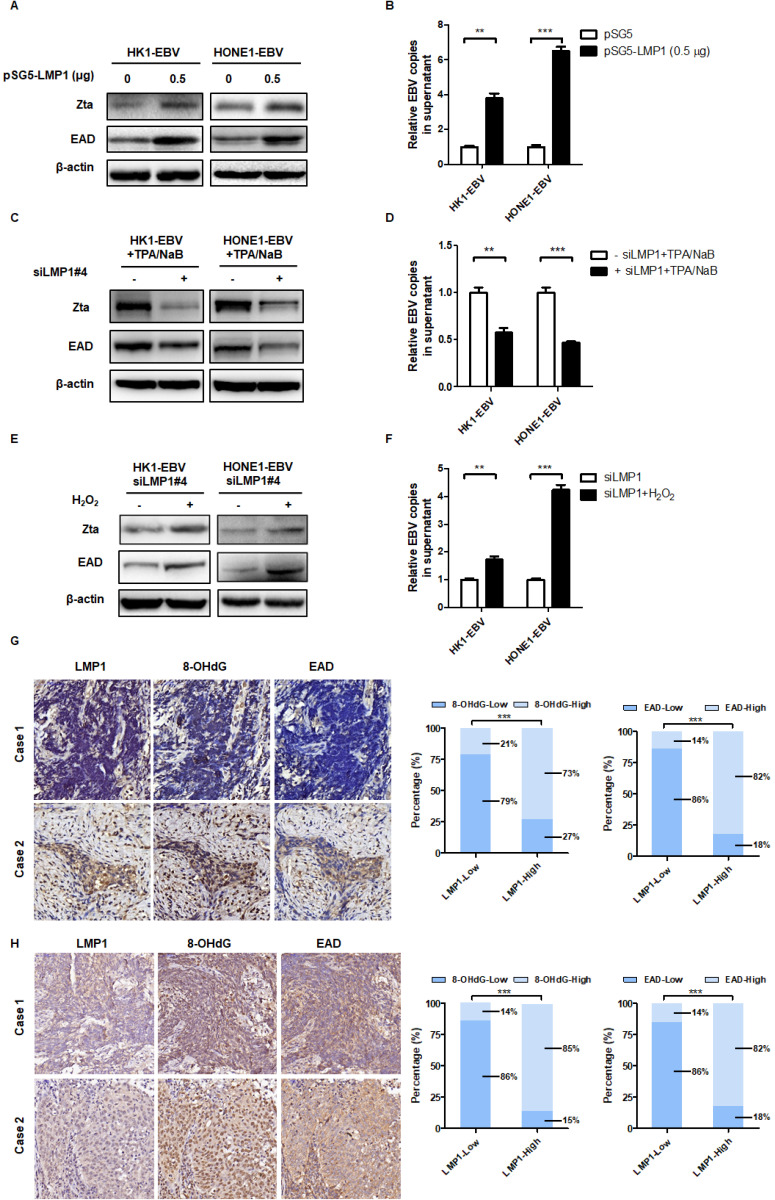
** LMP1 promotes EBV reactivation through the production of ROS. (A-B)** LMP1 overexpression induces EBV reactivation. HK1-EBV and HONE1-EBV cells were transfected with pSG5 and pSG5-LMP1 plasmids and the marker of EBV reactivation were detected using the same methods as described in Figure 3. Data are shown as means ± S.D.; n = 3; **p < 0.01, ***p < 0.001. **(C-D)** HK1-EBV and HONE1-EBV cells were transfected with LMP1 siRNA pool or negative siRNA, and induced by TPA/NaB treatment. The marker of EBV reactivation were detected. Data are shown as means ± S.D.; n = 3; **p < 0.01, ***p < 0.001. **(E-F)** LMP1 promotes EBV reactivation by production of ROS. HK1-EBV and HONE1-EBV cells were transfected with LMP1 siRNA pool, then H_2_O_2_ (50 µM, 24 h) was added to recover the ROS levels and then ROS and EBV reactivation levels were detected. Data are shown as means ± S.D.; n = 3; **p < 0.01, ***p < 0.001. **(G)** LMP1 is positively correlated with 8-OHdG and EAD in NPC patients. IHC analysis was used to examine the level of LMP1, 8-OHdG, and EAD in tumor biopsies from NPC patients. *(left)* Representative IHC staining of LMP1 and corresponding 8-OHdG and EAD expression is shown. *(right)* The expression of 8-OHdG and EAD was calculated based on LMP1 expression in NPC patients (***p < 0.001). **(H)** IHC analysis was used to examine the level of LMP1, 8-OHdG, and EAD in tumor tissue microarrays. *(left)* Representative IHC staining of LMP1 and corresponding 8-OHdG and EAD expression is shown. *(right)* The expression of 8-OHdG and EAD was calculated based on LMP1 expression in NPC patients (***p < 0.001).

Recently, our group and others found that LMP1 may have new functions in EBV reactivation 44-46. In order to determine the role of LMP1 in EBV lytic reactivation, we transfected an LMP1 overexpressing plasmid into HK1-EBV and HONE1-EBV cells and then examined the role of LMP1 in EBV reactivation. The results showed that LMP1 significantly induced EBV reactivation (Figure 4A-B). Then we transfected cells with LMP1 siRNA pool or negative siRNA and determined the role of LMP1 on EBV reactivation. Due to the low level of spontaneous lytic reactivation of EBV was observed in HK1-EBV and HONE1-EBV positive cell lines, the cells were transfected with LMP1 siRNA pool or negative control then followed by the treatment with TPA/NaB. The results showed that LMP1 depletion decreased EBV reactivation (Figure 4C-D). More importantly, in order to investigate whether LMP1 promotes EBV reactivation by production of ROS, EBV-positive cells were transfected with *LMP1* siRNA and then treated with H_2_O_2_ to recover ROS levels. The ROS level was shown in Figure S4 G-H. We found that LMP1 knockdown decreased EBV reactivation whereas the level of EBV reactivation was recovered by the addition of H_2_O_2_ (Figure 4E-F). These data illustrated that LMP1 promotes EBV reactivation through the production of ROS. Next, we detected the expression of LMP1, 8-OHdG, and EAD in NPC tumor slices and tumor tissue microarrays. The results showed that LMP1 is positively correlated with 8-OHdG and EAD in NPC patients (Figure 4G-H, Table 2 and Table 3).

In brief, these data supported the contention that LMP1 promotes EBV reactivation through production of ROS and LMP1 is positively correlated with oxidative stress and EBV reactivation in biopsies of NPC patients.

### High oxidative stress and high EBV reactivation are positively associated with poor survival of NPC patients following radiation therapy

**Figure 5:**
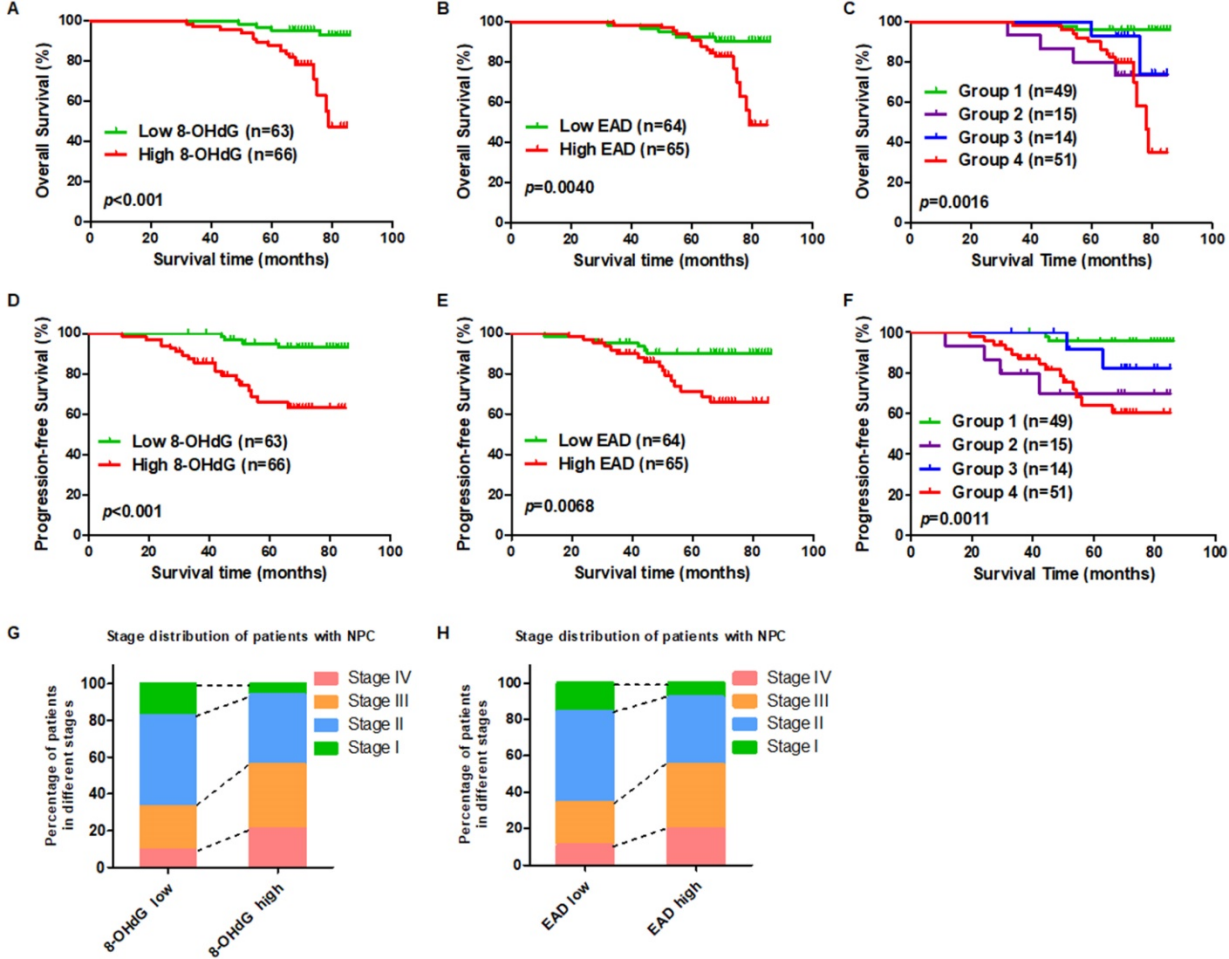
** High oxidative stress and high EBV lytic reactivation are positively associated with poor survival in NPC patients. (A)** A high expression level of 8-OHdG is associated with poor overall survival in NPC after radiation therapy. Overall survival rates of NPC patients after radiation therapy with low (n = 63) or high (n = 66) expression levels of 8-OHdG were estimated using the Kaplan-Meier method by log-rank test (*p* < 0.001). **(B)** A high expression level of EAD is associated with poor overall survival in NPC after radiation therapy. Overall survival rates of NPC patients after radiation therapy with low (n = 64) or high (n = 65) expression levels of EAD were estimated using the Kaplan-Meier method by log-rank test (*p* = 0.0040). **(C)** Cumulative overall survival curves of the combination of 8-OHdG and EAD. NPC patients were classified into 4 groups based on 8-OHdG and EAD expression: Group 1 (n = 49): low 8-OHdG and EAD expression; Group 2 (n = 15): high 8-OHdG but low EAD expression; Group 3 (n = 14): low 8-OHdG but high EAD expression; Group 4 (n = 51): high 8-OHdG and EAD expression. (*p* = 0.0016). **(D-F)** Progression-free survival analysis according to 8-OHdG and EAD expression. **(G)** The stage distribution of patients with NPC according to the level of 8-OHdG. **(H)** The stage distribution of patients with NPC according to the level of EAD.

To investigate the prognostic role of oxidative stress and EBV reactivation, two commercial NPC tumor tissue microarrays were used. According to the IHC scores for 8-OHdG and the EBV reactivation marker, EAD, we set the median score as the cut-off value and divided patients into two groups. The patients with high expression of 8-OHdG (n = 66; median survival time: 69 months *vs.* 81 months) had shorter overall survival compared to patients expressing lower levels of 8-OHdG (Figure 5A). Also, patients with high expression of EAD (n = 65; median survival time: 69 months *vs.* 81 months) had shorter overall survival compared to patients expressing lower levels of EAD (Figure 5B). Furthermore, to determine whether the combination of 8-OHdG and EAD could be used as a prognostic factor in predicting the outcome of EBV-positive NPC, patients were classified into 4 groups based on 8-OHdG and EAD expression: group 1 (n = 49): low 8-OHdG and EAD expression; group 2 (n = 15): high 8-OHdG but low EAD expression; group 3 (n = 14): low 8-OHdG but high EAD expression; group 4 (n = 51): high 8-OHdG and EAD expression. Differences in overall survival were significant among the 4 groups and group 4 had the worst prognosis (Figure 5C). More importantly, we also analyzed the patients' progression-free survival according to the level of 8-OHdG and EAD. The results showed that the NPC patients with a high level of 8-OHdG and EAD exhibited shorter progression-free survival than the patients with lower levels 8-OHdG and EAD and the group 4 which has high levels 8-OHdG and EAD had the worst prognosis (Figure 5D-F). In addition, we explored the stage distribution of patients with NPC according to the level of 8-OHdG and EAD. The results showed that the patients in stage III and IV has high levels of 8-OHdG and EAD while stage I and II has low levels of 8-OHdG and EAD (Figure 5G-H). Also, both 8-OHdG and EAD levels had no relationship with patients' age, sex, or neck lymph node but had significant relationship with patients' recurrence (Table S8 and Table S9). Another NPC tumor tissue microarray also confirmed the same conclusion (Figure S5). And both 8-OHdG and EAD levels had no relationship with patients' age, sex, or neck lymph node (Table S10 and Table S11).

These observations suggested that high oxidative stress and high EBV reactivation are associated with a worse clinical outcome of radiation therapy in NPC, which also means the high oxidative stress and EBV reactivation led to resistance of NPC patients to radiation therapy.

### NAC increases the sensitivity of NPC cells and tumors to radiation therapy both *in vitro* and *in vivo*

In order to explore the role of EBV in radioresistance, EBV negative and positive cells were treated with different doses of irradiation and the survival fraction was analyzed. The results showed that EBV positive cells were resistant to radiation therapy compared to EBV negative cells (Figure S6).

**Figure 6:**
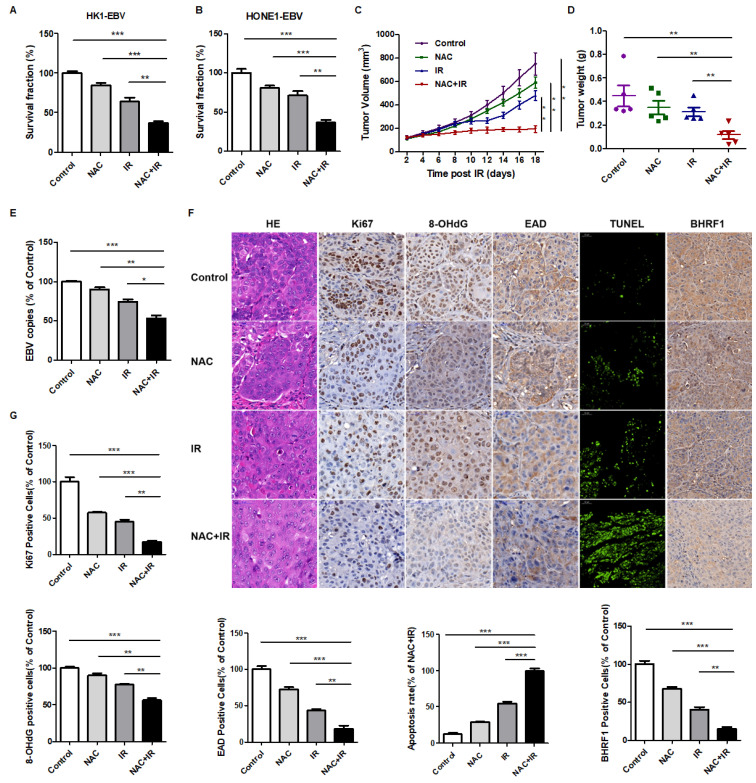
** NAC increases the radiosensitivity of NPC cells and tumors to radiation both *in vitro* and *in vivo.* (A-B)** NAC sensitizes HK1-EBV and HONE1-EBV cells to radiation therapy. Colony formation assay showing survival fractions of groups. Control: saline vehicle; NAC: NAC, 5 mM; IR: 4 Gy of irradiation restricted to cells; NAC + IR: NAC-irradiation combination. Surviving fractions were calculated by comparing the colony number of each treatment group with the control group. Results are plotted as the mean surviving fraction (means ± S.D. of 3 independent experiments performed; **p < 0.01, ***p < 0.001). **(C)** The growth curves of HONE1-EBV xenografts from the indicated treatment groups. Control: saline vehicle; NAC: NAC, 150 mg/kg, every two days, 3 weeks; IR: 2 Gy of irradiation restricted to tumors, twice a week, 2 weeks; NAC + IR: NAC-irradiation combination. Results are plotted as means ± S.D. (n = 5, each group; **p < 0.01). **(D)** Tumor weight was measured at the end of the experiments. Results are plotted as means ± S.D. (n = 5, each group; **p < 0.01). **(E)** EBV DNA in tumor xenografts was detected by using an EBV fluorescence quantitative PCR diagnostic kit. (n = 5, each group; *p < 0.05, **p < 0.01, ***p < 0.001). **(F)** Tumor sections were stained with hematoxylin and eosin (H&E) or subjected to immunohistochemistry detection for Ki67, 8-OHdG, EAD and BHRF1 (scale bar, 50 µm). Apoptosis of tumor sections was stained by TUNEL assay (scale bar, 100 µm). **(G)** Ki67, 8-OHdG, EAD, BHRF1, and TUNEL-positive cells were calculated. Data are shown as means ± S.D. **p < 0.01, ***p < 0.001.

Then as indicated earlier, EBV-induced high oxidative stress lead to radioresistance of NPC patients. Based on this hint, we determined whether the antioxidant, N-acetyl-L-cysteine (NAC), a U.S. Food and Drug Administration (FDA) approved drug, could lower the level of oxidative stress thereby enhance the sensitivity of NPC patients to radiation therapy. *In vitro* and *in vivo* experiments were performed. We treated EBV-positive cells with NAC, radiation (IR), or a combination of NAC and IR. A colony formation assay was used to evaluate the radiation resistance of cells. The results showed that NAC markedly increases the radiosensitivity of NPC cells (Figure 6A-B).

Next, to further confirm this finding *in vivo*, we treated athymic nude mice bearing HONE1-EBV xenografts with NAC, radiation (IR), or a combination of NAC and IR. Consistent with the *in vitro* results, NAC combined with radiation significantly reduced tumor growth (Figure 6C-D). EBV copy number in tumor cells is an important marker that indicates the infection and reactivation of EBV. Therefore, we also detected EBV copy number in tumor samples. We found that as shown in Figure 6E, the EBV copy number was significantly decreased in the NAC + IR group compared with other groups. These results indicated that NAC sensitizes HONE1-EBV xenografts to radiation. In addition, IHC experiments showed less Ki67 staining, which is indicative of reduced proliferation. The 8-OHdG and EAD stained cells also decreased in the combined therapy groups, which indicated that the oxidative stress and EBV lytic reactivation levels were inhibited. Moreover, more TUNEL positive cells were present in NAC + IR tumors, suggesting that the combined therapy represses cell growth in xenografts through the induction of apoptosis. Thus, the decrease of the EBV-encoded anti-apoptotic protein, BHRF1, might play a vitally important role in this process (Figure 6F-G).

Overall, both *in vitro* and *in vivo* data demonstrated that NAC could increase the sensitivity of NPC to radiation through decreased oxidative stress, which shows a promising prospect in NPC clinical therapy.

### 8-OHdG and EBV DNA could be dual markers for NPC patients

**Figure 7:**
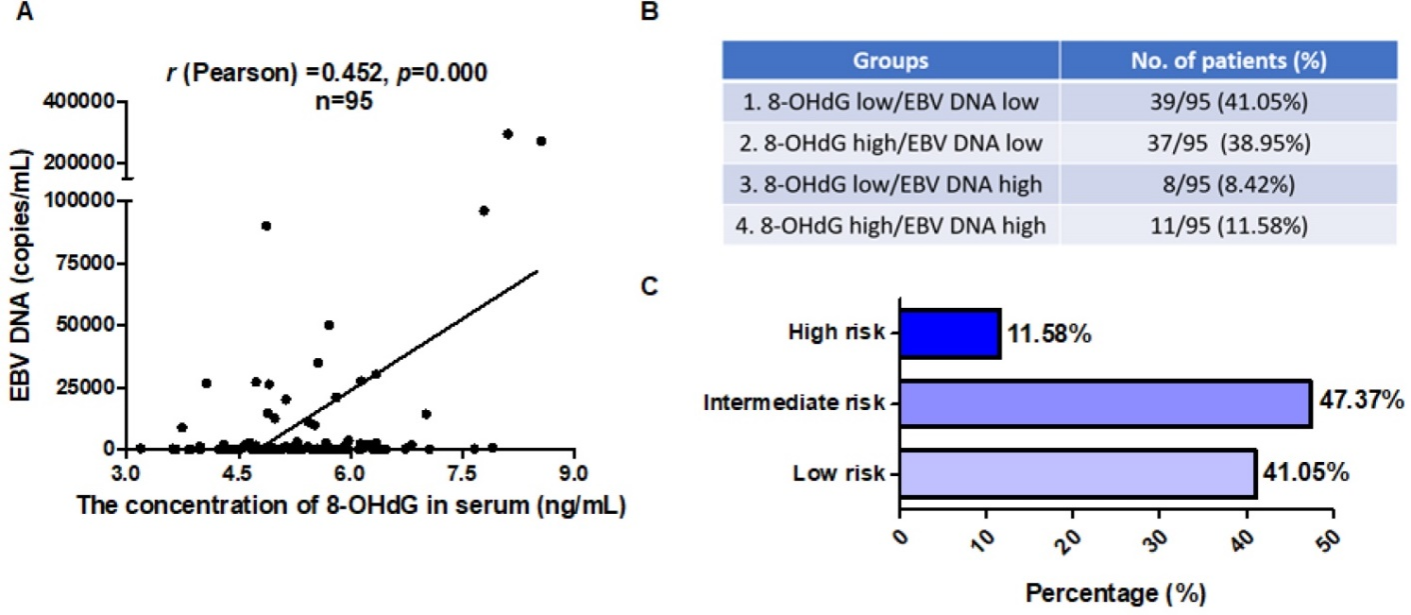
** 8-OHdG and EBV DNA could be prognostic markers for NPC patients. (A)** Serum 8-OHdG concentrations in NPC patients were assessed using ELISA method. EBV DNA copies in serum of the corresponding NPC patients (n = 95) were detected by using an EBV fluorescence quantitative PCR diagnostic kit. The correlation between 8-OHdG and EBV DNA is shown by scatter plot; *r* (Pearson) = 0.452, *p* = 0.000. **(B)** Generation of a molecular subtyping model for NPC based on 8-OHdG and EBV DNA level. The percentage of 4 groups was calculated based on the level of 8-OHdG and EBV DNA. EBV DNA cut-off value was set as 4,000 copies/mL based on clinical guidance. The median score of 8-OHdG was set as cut-off value and divided patients into high and low groups. **(C)** Patients were classified into 3 risk groups based on 8-OHdG and EBV DNA level.

Over the last decade, EBV DNA has been developed as a tumor marker for NPC. Plasma EBV DNA analysis using real-time PCR has been widely used in early detection, prognostication, and monitoring of treatment response of NPC 15,26,47-50. Thus, we next investigated whether 8-OHdG and the combination of 8-OHdG and EBV DNA could be prognostic markers for NPC. First, to elucidate the clinical relevance of 8-OHdG and EBV DNA, their levels were analyzed in clinical NPC samples. Statistical analysis of the data demonstrated a significant positive correlation between these markers in NPC samples (*r* (Pearson) = 0.452; Figure 7A). Next, in order to determine the ratio of high EBV DNA and high oxidative stress, NPC patients were then grouped based on the expression levels of 8-OHdG and EBV DNA. EBV DNA cut-off value was set as 4,000 copies/mL based on clinical guidance. The median score of 8-OHdG was set as the cut-off value and patients were divided into two groups. To determine whether the combination of 8-OHdG and EBV DNA could be used as prognostic factors in predicting the outcome of NPC, patients were classified into 4 groups based on 8-OHdG and EBV DNA expression: group 1 (39/95): low 8-OHdG and EBV DNA expression; group 2 (37/95): high 8-OHdG but low EBV DNA expression; group 3 (8/95): low 8-OHdG but high EBV DNA expression; and group 4 (11/95): low 8-OHdG and EBV DNA expression (Figure 7B). Group 4 exhibited high radioresistance and thus patients might benefit from antioxidant therapy (Figure 7C). Based on these results, we, for the first time, generate a molecular subtyping model for NPC relying on 8-OHdG and EBV DNA levels, thereby providing novel perspectives for the precise treatment of NPC.

## Discussion

Radiation resistance has been a major hurdle for local control of cancers by radiotherapy. Our present study demonstrated that EBV induces high oxidative stress, which promotes its reactivation, thus leading to cancer radioresistance both evidenced in NPC cells and clinical samples including serum and NPC biopsies. Moreover, by decreasing the oxidative stress using an FDA approved drug like NAC could inhibit EBV reactivation and thus sensitize NPC to radiation therapy, offering opportunities for novel therapeutic intervention. More importantly, high oxidative stress and EBV reactivation were associated with poor survival in NPC patients. Thus, we, for the first time, proposed a molecular subtyping model for NPC relying on dual markers 8-OHdG and EBV DNA. This is very convenient to detect and identify patients at a high risk for poor outcomes, suggesting that these patients might benefit from antioxidant therapy. Therefore, this discovery presents a new and promising strategy for NPC patients exhibiting high oxidative stress.

Cancer cells are clearly characterized by high levels of oxidative stress 51. Generating oxidative stress is a critical mechanism by which host cells defend against infection by pathogenic microorganisms 52. Based on the promoting role of ROS in cancer progression, antioxidant therapy represents one of the treatments for cancer therapy 39,43. Some patients have benefit from the therapy, but some of them are failed. The reasons have been summarized as follows 53. First, many agents that are called antioxidants are truly antioxidants at a given dose, but this dose may not have been given in clinical trials. Second, many agents are not antioxidants at all. Third, not all tumors use reactive oxygen as a signaling mechanism. Finally, reactive oxygen inhibition is often insufficient to kill or regress a tumor cell by itself, but requires sequential introduction of a therapeutic agent for maximal effect. Thus, a concept of ROS-driven tumors was proposed. So far, several types of tumors have been classified into reactive oxygen species (ROS)-driven tumors and include EBV-associated Burkitt's lymphoma, Hodgkin's disease, gastric carcinoma, hepatitis C virus-related hepatocellular carcinoma, ultraviolet A-induced melanoma, inflammatory bowel disease-induced colon carcinoma, schistosomiasis-induced bladder cancer, tobacco-induced oral and lung carcinoma, and epidermolysis bullosa-associated squamous cell carcinoma 17,53. These cancers are characterized by activation of high expression levels of NF-κB, Akt, wild-type p53 and PTEN 53. In this study, we demonstrated that both EBV-positive NPC cells including EBV latent infection and primary infection and patients exhibited high levels of oxidative stress, which is consistent with the reports of Segawa et al 54. Mechanistically, EBV not only upregulates the expression of ROS-producing enzyme, NOX2, and but also upregulates the cellular antioxidant master regulator, Nrf2, to combat the overproduction of ROS to induce cell death.

Radiotherapy is the preferred treatment for NPC; however, recurrence often occurs because tumor cells become resistant to radiation 27,55,56. Presently, targeting several biomarkers have been identified to enhance the radiosensitivity of NPC such as Bcl-2, VEGF, EGFR and AMPK which were associated with cell apoptosis, angiogenesis, cell cycle and DNA repair. In our previous study, we demonstrated that the EBV-encoded driving oncogene, *LMP1,* contributes to the radioresistance of NPC. LMP1 appears to activate several oncogenic signaling axes, including LMP1/JNKs/c-Jun/HIF-1/VEGF 32, LMP1/NF-κB/ATM 57, LMP1/Akt/hTERTs 58, LMP1/PI3K/Akt/GSK3β/c-Myc/HK2 29, and LMP1/DNA-PK/AMPK/DDR 31, which mediate therapeutic resistance. Here, we found that reactivation of EBV by LMP1-induced high oxidative stress could lead to radioresistance of NPC cells both *in vitro* and *in vivo*, suggesting that EBV reactivation plays an important role in NPC resistance, and LMP1 is one of the factors that mediated resistance.

Oxidative stress plays an important role in EBV infection. Previous studies have shown that several compounds such as chlorpyrifos 59, chaetocin 60, N-Methyl-N'-Nitro-N-Nitrosoguanidine (MNNG) 61 can induce EBV lytic reactivation via ROS. Especially, p53-dependent mechanism has been proposed to be related to ROS-mediated EBV reactivation 61. These reports revealed the critical role of ROS in mediating the switch of EBV from the latent to the lytic phase. In addition, ROS-dependent signaling is correlated with the oxidation of specific proteins 62. In our work, we found that high oxidative stress promotes EBV reactivation and that the oxidative stress level was indeed positively correlated with EBV reactivation both *in vitro* and* in vivo*. The possible mechanism involved in the regulation of EBV reactivation by oxidative stress may also correspond with the oxidative modification of specific proteins directly or indirectly, which needs to be further studied.

In NPC clinical therapy, accumulating evidence regarding EBV DNA has emphasized its crucial role in diagnosis, prognosis, and prediction of the therapeutic response of NPC 8,15,26,27,47-50,63-66. The higher EBV DNA levels predict poor survival of NPC patients. Here, we confirmed that patients exhibiting high oxidative stress and EBV reactivation exhibited poor overall survival and progression-free survival. Also, the patients in stage III and IV has high levels of 8-OHdG and EAD while stage I and II has low levels of 8-OHdG and EAD. Our proposed molecular subtyping model is based on oxidative stress and EBV DNA, which are dual markers that could accurately stratify patients at high risk. Notably, those patients might be benefit from the combined therapy of antioxidants and radiation, thereby providing novel perspectives for the precise treatment of NPC.

In summary, we demonstrated EBV induces high oxidative stress, which promotes EBV reactivation and leads to therapeutic radioresistance. Furthermore, high oxidative stress and EBV reactivation were associated with poor survival outcome of NPC patients. All these conclusions were validated in cells, xenograft tumors, and clinical patient samples, including serum and biopsies. More importantly, we, for the first time, proposed a molecular subtyping model based on dual markers of oxidative stress and EBV DNA and confirmed antioxidant therapy as an effective treatment for patients with NPC at high risk (i.e., with high oxidative stress and high EBV DNA levels). This could facilitate the development of novel predictive and therapeutic strategies against radiation-resistant NPC.

## Supplementary Material

Supplementary figures and tables.

## Figures and Tables

**Figure 8:**
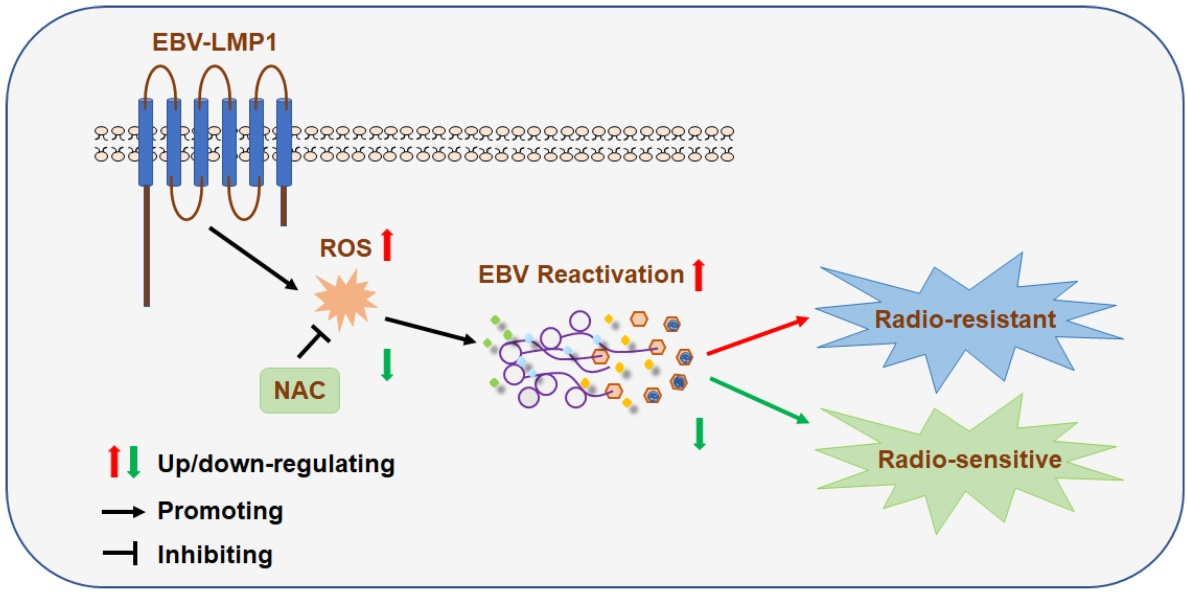
** A schematic to illustrate EBV (LMP1)-mediated radiation resistance in nasopharyngeal carcinoma (NPC).** EBV (LMP1) induces high oxidative stress, which promotes EBV reactivation and leads to therapeutic radioresistance.

**Table 1:** Correlation between 8-OHdG and EAD in 92 NPC patients

			EAD	
Biopsies	Spearman's rho	8-OHdG	Correlation coefficient	0.437
			Significance (2-tailed)	0.000
			N	92

**Table 2:** Correlation among LMP1, 8-OHdG and EAD in 25 NPC patients

				8-OHdG	EAD
Biopsies	Spearman's rho	LMP1	Correlation coefficient	0.619	0.763
			Significance (2-tailed)	0.001	0.000
			N	25	25

**Table 3:** Correlation among LMP1, 8-OHdG and EAD in 129 NPC patients

				8-OHdG	EAD
Biopsies	Spearman's rho	LMP1	Correlation coefficient	0.768	0.843
			Significance (2-tailed)	0.000	0.000
			N	129	129

## Abbreviations

BL: Burkitt's Lymphoma
EBV: Epstein-Barr Virus
EBNA1: Epstein-Barr nuclear antigen 1
ELISA: Enzyme-linked immunosorbent assay
FBS: fetal bovine serum
FCM: flow cytometry
FDA: Food and Drug Administration
HBV: hepatitis B virus
HE: hematoxylin and eosin
HIV: humam immunodeficiency virus
HSV-1: herpes simplex virus 1
IHC: immunohistochemistry
IMRT: intensity-modulated radiotherapy
KSHV: Kaposi's sarcoma-associated herpesvirus
LMP1: latent membrane protein 1
NAC: N-acetyle-L-cysteine
NADPH: nicotinamide adenine dinucleotide phosphate hydrogen
NP: nasopharyngitis patients
NPC: nasopharyngeal carcinoma
Nrf2: nuclear actor erythroid 2-related-factor 2
NOX2: NADPH oxidase 2
PCR: polymerase chain reaction
ROS: reactive oxygen species
RT-qPCR: real-time quantitative polymerase chain reaction
TUNEL: terminal deoxynucleotidyl transferase-mediated Dutp nick-end labeling
VEGF: vascular endothelial growth factor
8-OHdG: 8-hydroxydeoxyguanosine
