# Targeting Epstein-Barr virus oncoprotein LMP1-mediated high oxidative stress suppresses EBV lytic reactivation and sensitizes tumors to radiation therapy: Erratum

**DOI:** 10.7150/thno.73630

**Published:** 2022-04-26

**Authors:** Jianmin Hu, Yueshuo Li, Hongde Li, Feng Shi, Longlong Xie, Lin Zhao, Min Tang, Xiangjian Luo, Weihua Jia, Jia Fan, Jian Zhou, Qiang Gao, Shuangjian Qiu, Weizhong Wu, Xin Zhang, Weihua Liao, Ann M. Bode, Ya Cao

**Affiliations:** 1 Key Laboratory of Cancer Carcinogenesis and Invasion, Chinese Ministry of Education, Xiangya Hospital, Central South University, Changsha 410078, China.; 2 Cancer Research Institute and School of Basic Medicine Science, Xiangya School of Medicine, Central South University, Changsha 410078, China.; 3 Key Laboratory of Carcinogenesis, Chinese Ministry of Health, Changsha 410078, China.; 4 Molecular Imaging Research Center of Central South University, Changsha 410078, China.; 5 Research Center for Technologies of Nucleic Acid-Based Diagnostics and Therapeutics, Changsha 410078, China.; 6 National Joint Engineering Research Center for Genetic Diagnostics of Infectious Diseases and Cancer, Changsha 410078, China.; 7 State Key Laboratory of Oncology in South China, Collaborative Innovation Center for Cancer Medicine, Guangdong Key Laboratory of Nasopharyngeal Carcinoma Diagnosis and Therapy, Sun Yat-sen University Cancer Center, Guangzhou, 510060, China.; 8 Key Laboratory of Cancer Carcinogenesis and Invasion, Chinese Ministry of Education, Zhongshan Hospital, Shanghai Medical School, Fudan University, Shanghai 200000, China.; 9 Department of Otolaryngology Head and Neck Surgery, Xiangya Hospital, Central South University, Changsha 410078, China.; 10 Department of Radiology, Xiangya Hospital, Central South University, Changsha 410078, China.; 11 The Hormel Institute, University of Minnesota, Austin, MN 55912, USA.

In the original publication, errors were found in Fig. 4H (right). The images were misassigned. We re-analyzed the raw data and correct the images. The correct figures are shown below. The authors confirm that these corrections do not change the result interpretation or conclusions of the article. The authors are sorry and sincerely apologize for any inconvenience or misunderstanding that may have caused.

## Figures and Tables

**Figure 4:**
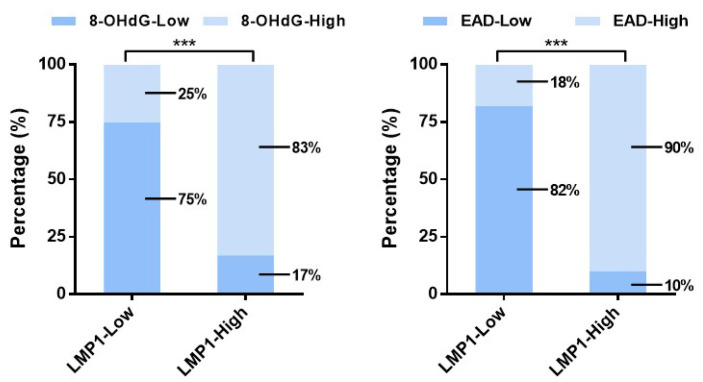
**H** (right) The expression of 8-OHdG and EAD was calculated based on LMP1 expression in NPC patients (***p < 0.001).

