# Supplementary material for: Targeting Epstein-Barr virus oncoprotein LMP1-mediated high oxidative stress suppresses EBV lytic reactivation and sensitizes tumors to radiation therapy
Source: Theranostics. 2020 Oct 25;10(26):11921–37. doi: 10.7150/thno.46006 (PMC7667690; doi:10.7150/thno.46006)
Supplement: Supplementary file 1 — Supplementary figures and tables. [file thnov10p11921s1.pdf]

Supplemental Materials

Table S1. The clinical characteristics of 95 patients with primary NPC diagnosis

Characteristics	No. of patients (%)
**Age, year (Range 15-84)**	
< 47	46 (48.4)
≥ 47	49 (51.6)
**Gender**	
Male	75 (78.9)
Female	20 (21.1)
**T classification**	
T1, T2	12 (12.6)
T3, T4	83 (87.4)
**N classification**	
N0	5 (5.3)
N1, N2, N3	90 (94.7)
**M classification**	
M0	88 (92.6)
M1	7 (7.4)
**Clinical stage**	
Ⅱ	12 (12.6)
Ⅲ	42 (44.2)
Ⅳ	41 (43.2)

**WHO histological classification**	
NKUC(UD)	88 (92.6)
NKUC(DF)	2 (2.1)
Others	5 (5.3)

Table S2. The clinical characteristics of 8 NP and 25 NPC patients

	Clinical characteristics	No. of patients (%)
**NP**	**Age, year (Range 30-69)**	
	< 49	3 (37.5)
	≥ 49	5 (62.5)
	**Gender**	
	Male	5 (62.5)
	Female	3 (37.5)
**NPC**	**Age, year (Range 35-73)**	
	< 48	9 (36.0)
	≥ 48	16 (64.0)
	**Gender**	
	Male	16 (64.0)
	Female	9 (36.0)
	**WHO histological classification**	
	NKUC(UD)	21 (84.0)
	NKUC(DF)	4 (16.0)

Table S3. The clinical characteristics of 30 NP and 92 NPC patients assessed by microarray

	Clinical characteristics	No. of patients (%)
**NP**	**Age, year (Range 16-72)**	
	< 39	14 (46.7)
	≥ 39	16 (53.3)
	**Gender**	
	Male	18 (60.0)
	Female	12 (40.0)
**NPC**	**Age,year (Range 25-68)**	
	< 45	44 (47.8)
	≥ 45	48 (52.2)
	**Gender**	
	Male	71 (77.2)
	Female	21 (22.8)
	**Neck lymph node**	
	Negative	37 (40.2)
	Positive	40 (43.5)
	No information	15 (16.3)
	**EBER status**	
	Negative	0

	Positive	92 (100)

Table S4. The clinical characteristics of 129 NPC patients assessed by microarray

Clinical characteristics	No. of patients (%)
**Age, year (Range 20-82)**	
≤ 47	67 (51.9)
> 47	62 (48.1)
**Gender**	
Male	100 (77.5)
Female	29 (22.5)
**Neck lymph node**	
Negative	35 (27.1)
Positive	94 (72.9)
**Recurrence**	
Negative	70 (54.3)
Positive	58 (45.0)
No information	1 (0.7)
**EBER**	
Negative	2 (1.5)
Positive	127 (98.5)
**Clinical stage**	
I	15 (11.6)

II	56 (43.4)
III	38 (29.5)
IV	20 (15.5)

Table S5. SiRNA sequence

siRNA	Sequence
**siNOX2 #1**	GGCCCAACTGGGATAATGA
**siNOX2 #2**	GTCCCATGTTTCTGTATCT
**siLMP1 #3**	CAUUGUUCCUUGGAAUUGUTT
**siLMP1 #4**	GGUAUCUGGAUCUACUUAUTT

Table S6. Primer sequences used in this study

Gene	Forward Primer sequence	Reverse primer sequence
**LMP1**	CGTTATGAGTGACTGGACTGGA	TGAACAGCACAATTCCAAGG
**BARF1**	CAGGTTCATCGCTCAGCTCC	CATGGGAGATGTTGGCAGC
**EBER1**	AGGACCTACGCTGCCCTAGA	AAAACATGCGGACCACCAGC
**BZLF1**	CATGTTTCAACCGCTCCGACTGG	GCGCAGCCTGTCATTTTCAGATG
**BMRF1**	CTAGCCGTCCTGTCCAAGTGC	AGCCAAACGCTCCTTGCCCA
**NOX2**	CCCTTTGGTACAGCCAGTGAAGAT	CAATCCCAGCTCCCACTAACATCA
**Nrf2**	TACTCCCAGGTTGCCCACA	CATCTACAAACGGGAATGTCTGC
**NQO1**	CATTCTGAAAGGCTGGTTTGA	CTAGCTTTGATCTGGTTGTCAG
**SOD1**	CTGAAGGCCTGCATGGATTC	CCAAGTCTCCAACATGCCTCTC
**XDH**	AGGTGGACCACTTCAGCAAT	GTTGGAGGGAAGGTTGGTTT

**GPX7**	CCCACCACTTTAACGTGCTC	GGCAAAGCTCTCAATCTCCTT
**GPX8**	CCGCCCAAGCAAGGAAGTAG	TCTAACCAGAGCTGC TATGTCAG
**β-actin**	CCAAGGCCAACCGCGAGAAGATGAC	AGGGTACATGGTGGTGCCGCC AGAC
**BamHI-W region DNA**	GGGTGCAGTAACAGGTAATC	ATTCGCCTCTAAAGTTTTGA
**β-globin DNA**	CTGGCAAATTGGATAAGGAGTCA	TCCATCCTTTTATTTCGAGCC

Table S7. Correlation between 8-OHdG and EAD in 129 NPC patients

			EAD	
Biopsies	Spearman's rho	8-OHdG	Correlation coefficient	.725
			Significance (2-tailed)	.000
			N	129

Table S8. Clinical characteristics and 8-OHdG level in 129 non-keratinizing undifferentiated NPC patients who received radiation therapy

**Clinical characteristics**	**8-OHdG Low level**	**8-OHdG High level**	***p*-value**
**Age (yr),** mean±S.D.	49.33±10.95	46.94±11.83	0.374[a]
**Gender**			0.87[b]
Male (n)	51	49	
Female (n)	12	17	
**Neck lymph node**			0.349[b]
Negative (n)	20	15	
Positive (n)	43	51	

**Recurrence**			0.000[b]
Negative (n)	49	21	
Positive (n)	14	44	
No information (n)	0	1	
**Clinical Stage**			0.028[b]
Ⅰ/Ⅱ	42	21	
Ⅲ/Ⅳ	29	47	

[a] Welch’s test

[b] Pearson’s χ^2 text

Table S9. Clinical characteristics and EAD level in 129 non-keratinizing undifferentiated NPC patients who received radiation therapy

**Clinical characteristics**	**EAD Low level**	**EAD High level**	***p*-value**
**Age (yr)**, mean±S.D.	49.53±11.25	46.71±11.51	0.326[a]
**Gender**			0.558[b]
Male (n)	49	51	
Female (n)	15	14	
**Neck lymph node**			0.589[b]
Negative (n)	17	18	0.589[b]
Positive (n)	47	47	
**Recurrence**			0.000[b]

Negative (n)	45	25	
Positive (n)	19	39	
No information (n)	0	1	
**Clinical Stage**			0.011[b]
Ⅰ/Ⅱ	42	29	
Ⅲ/Ⅳ	22	36	

[a] Welch's test

[b] Pearson's χ^2 text

Table S10. Clinical characteristics and 8-OHdG level in 92 non-keratinizing undifferentiated NPC patients who received radiation therapy

**Clinical characteristics**	**8-OHdG Low level**	**8-OHdG High level**	***p*-value**
**Age (yr),** mean±S.D.	44.52±10.60	45.09±8.69	0.780[a]
**Gender**			
Male (n)	35	36	0.804[b]
Female (n)	11	10	
**Neck lymph node**			
Negative (n)	21	16	0.303[b]
Positive (n)	18	22	
No information (n)	7	8	

[a] Welch's test

[b] Pearson's χ^2 text

Table S11. Clinical characteristics and EAD level in 92 non-keratinizing undifferentiated NPC patients who received radiation therapy

Clinical characteristics	EAD Low level	EAD High level	*p*-value
**Age (yr)**, mean±S.D.	44.28±10.17	45.33±9.17	0.607[a]
**Gender**			
Male (n)	35	36	0.804[b]
Female (n)	11	10	
**Neck lymph node**			
Negative (n)	21	16	0.417[b]
Positive (n)	19	21	
No information (n)	6	9	

[a] Welch's test

[b] Pearson's χ^2 text

Supplemental Figures and Figure Legends

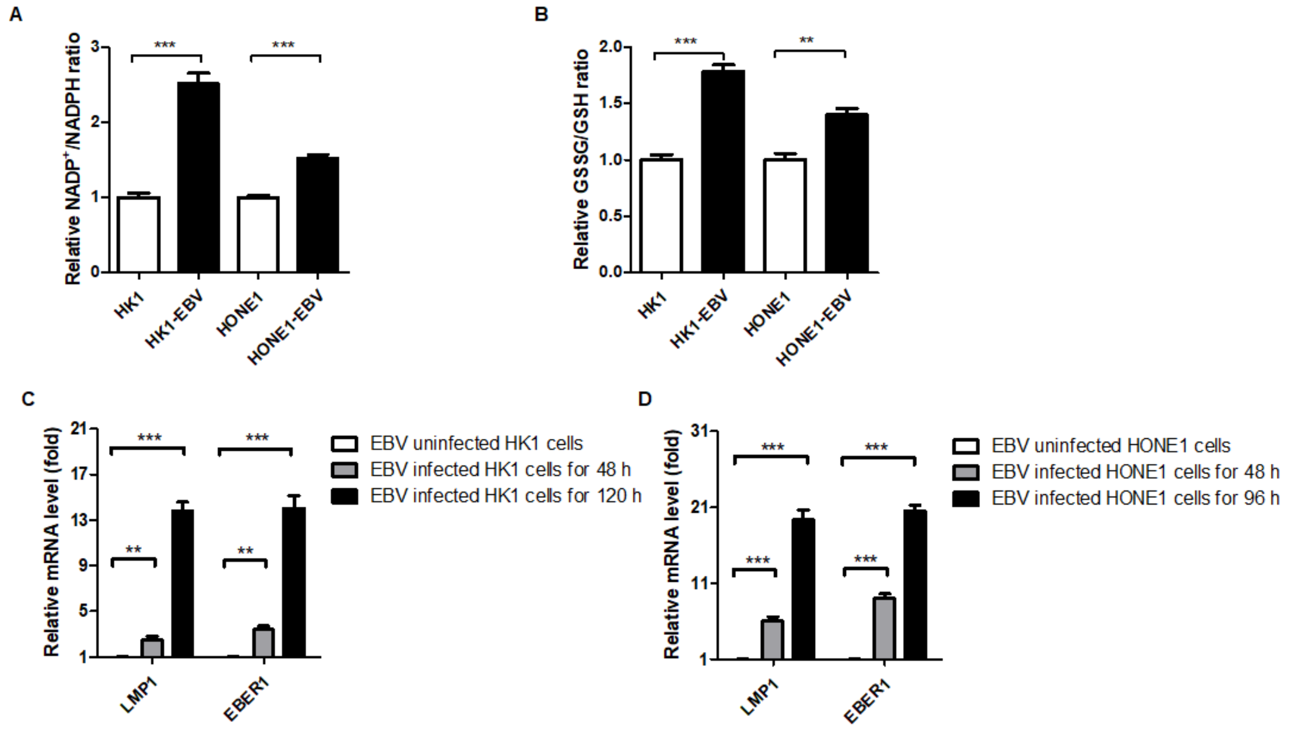

Figure S1. EBV induces high oxidative stress in NPC cells and patients. (A) Intracellular $NADP^+$/NADPH levels were assayed using an $NADP^+$/NADPH assay kit. Data are shown as means ± S.D.; n = 3; ***$p < 0.001$. **(B)** Total glutathione (GSH) and oxidized glutathione (GSSG) were measured using a GSH/GSSG assay kit. Data are shown as means ± S.D.; n = 3; **$p < 0.01$, ***$p < 0.001$. **(C)** The infection in HK1 cells was assessed by the detection of EBV genes (LMP1 and EBER1) by qPCR. Data are shown as means ± S.D.; n = 3; **$p < 0.01$, ***$p < 0.001$. **(D)** The infection in HONE1 cells was assessed by the detection of EBV genes (LMP1 and EBER1) by qPCR. Data are shown as means ± S.D.; n = 3; **$p < 0.01$, ***$p < 0.001$.

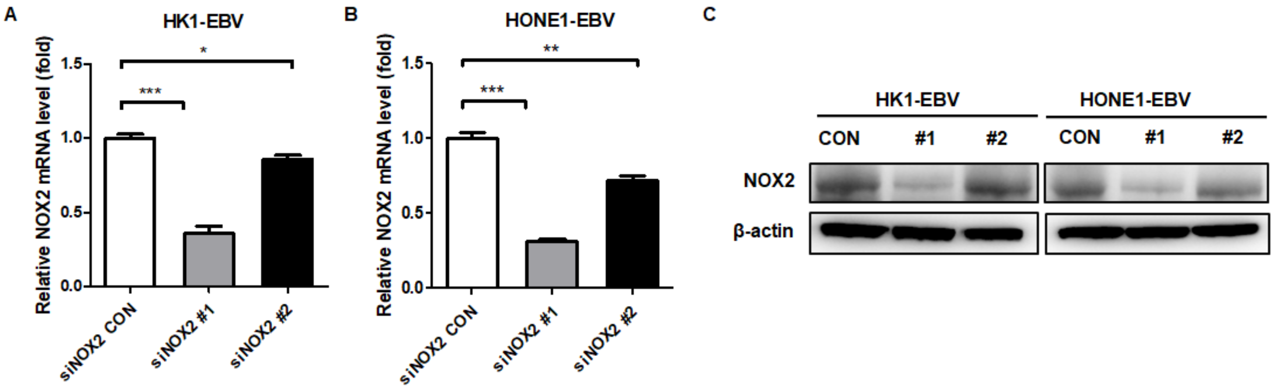

Figure S2. The NOX2 knockdown efficiency by siRNA transfection was detected by qPCR and Western blot. (A-B) The mRNA levels of *NOX2* after siRNA transfection were detected by RT-PCR. Data are shown as means ± S.D.; n = 3; **p* < 0.05, ***p* < 0.01, ****p* < 0.001. **(C)** NOX2 protein expression was detected by Western blot analysis and β-actin was used as an internal control.

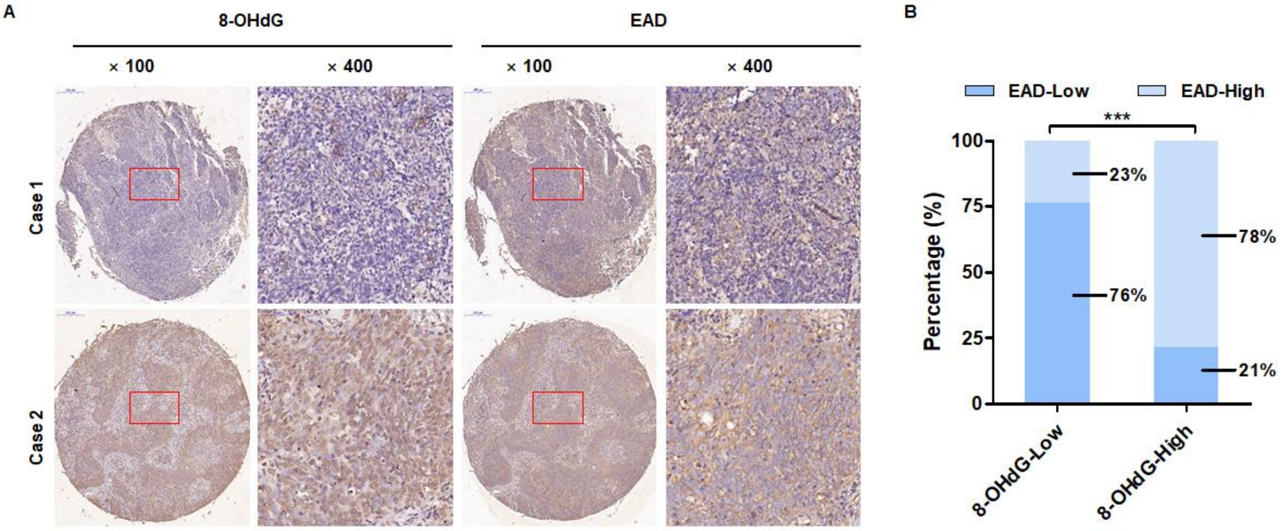

Figure S3. 8-OHdG expression was positively correlated with EAD in human NPC patients. (A) Representative IHC photographs showing the expression of 8-OHdG and EAD in consecutive sections of NPC microarrays (HNasN129Su01, Shanghai, China). **(B)** EAD level is calculated based on 8-OHdG expression in NPC microarrays (****p* < 0.001).

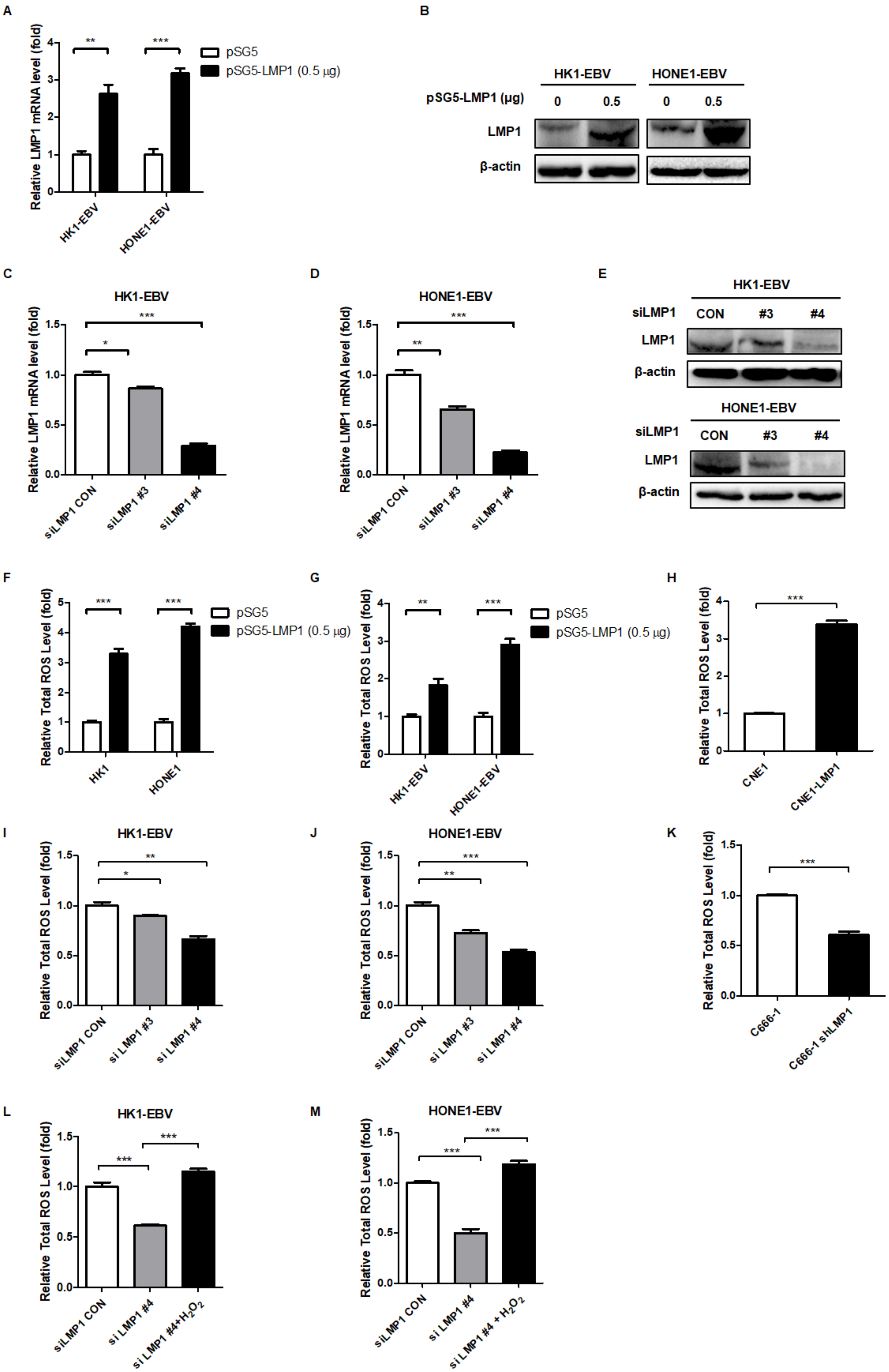

A
Relative LMP1 mRNA level (fold)
**
***
pSG5
pSG5-LMP1 (0.5 μg)
HK1-EBV
HONE1-EBV
B
HK1-EBV
HONE1-EBV
pSG5-LMP1 (μg)
0
0.5
0
0.5
LMP1
β-actin
C
HK1-EBV
Relative LMP1 mRNA level (fold)
*
***
siLMP1 CON
siLMP1 #3
siLMP1 #4
D
HONE1-EBV
Relative LMP1 mRNA level (fold)
**
***
siLMP1 CON
siLMP1 #3
siLMP1 #4
E
HK1-EBV
siLMP1
CON
#3
#4
LMP1
β-actin
HONE1-EBV
siLMP1
CON
#3
#4
LMP1
β-actin
F
Relative Total ROS Level (fold)
***
***
pSG5
pSG5-LMP1 (0.5 μg)
HK1
HONE1
G
Relative Total ROS Level (fold)
**
***
pSG5
pSG5-LMP1 (0.5 μg)
HK1-EBV
HONE1-EBV
H
Relative Total ROS Level (fold)
***
CNE1
CNE1-LMP1
I
HK1-EBV
Relative Total ROS Level (fold)
*
**
siLMP1 CON
si LMP1 #3
si LMP1 #4
J
HONE1-EBV
Relative Total ROS Level (fold)
**
***
siLMP1 CON
si LMP1 #3
si LMP1 #4
K
Relative Total ROS Level (fold)
***
C666-1
C666-1 shLMP1
L
HK1-EBV
Relative Total ROS Level (fold)
***
***
siLMP1 CON
si LMP1 #4
si LMP1 #4+H2O2
M
HONE1-EBV
Relative Total ROS Level (fold)
***
***
siLMP1 CON
si LMP1 #4
si LMP1 #4 + H2O2

Figure S4. LMP1 promotes the production of ROS. The LMP1 overexpression and knockdown efficiency was detected by qPCR and Western blot. **(A)** The mRNA levels of *LMP1* after pSG5-LMP1 plasmid transfection were detected by qPCR. Data are shown as means ± S.D.; n = 3; ***p* < 0.01, ****p* < 0.001. **(B)** LMP1 protein expression after pSG5-LMP1 plasmid transfection was detected by Western blot analysis and β-actin was used as an internal control. **(C-D)** The mRNA levels of *LMP1* after siLMP1 transfection were detected by qPCR. Data are shown as means ± S.D.; n = 3; **p* < 0.05, ***p* < 0.01, ****p* < 0.001. **(E)** LMP1 protein expression was detected by Western blot analysis and β-actin was used as an internal control. **(F-G)** LMP1 induces ROS generation. EBV cells were transfected with pSG5 and pSG5-LMP1 plasmids and total ROS levels were detected. Data are shown as means ± S.D.; n = 3; ***p* < 0.01, ****p* < 0.001. **(H)** The total ROS levels in CNE1 and LMP1 stable overexpression cell line CNE1-LMP1 were detected. Data are shown as means ± S.D.; n = 3; ****p* < 0.001. **(I-J)** LMP1 knockdown decreases ROS generation. HK1-EBV and HONE1-EBV cells were transfected with LMP1 siRNA pool and total ROS levels were detected. Data are shown as means ± S.D.; n = 3; **p* < 0.05, ***p* < 0.01, ****p* < 0.001. **(K)** The total ROS levels in C666-1 and LMP1 stable cell line C666-1 shLMP1 were detected. Data are shown as means ± S.D.; n = 3; ****p* < 0.001. **(L-M)** HK1-EBV and HONE1-EBV cells were transfected with LMP1 siRNA pool or negative siRNA, then H_2O_2 was added to recover the ROS levels and the ROS levels were detected. Data are shown as means ± S.D.; n = 3; ****p* < 0.001.

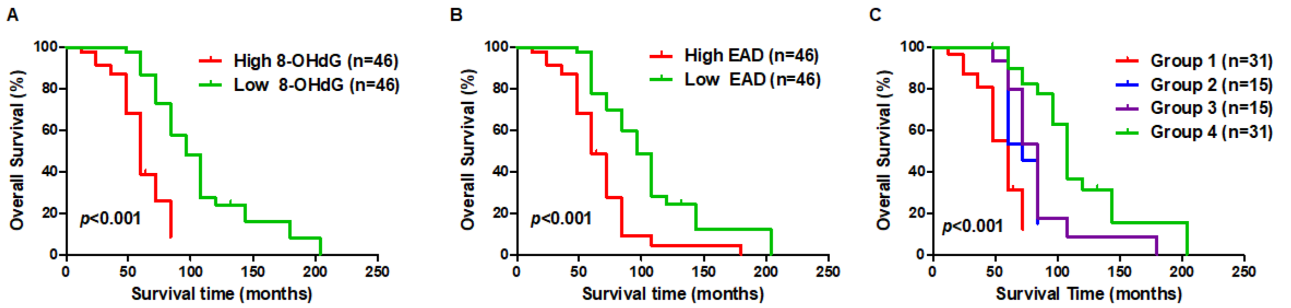

Figure S5. High oxidative stress and high EBV lytic reactivation are positively associated with poor survival in NPC patients. (A) A high expression level of 8-OHdG is associated with poor overall survival in NPC after radiation therapy. Overall survival rates of NPC patients after radiation therapy with low (n = 46) or high (n = 46) expression levels of 8-OHdG were estimated using the Kaplan–Meier method by log-rank test (p < 0.001). **(B)** A high expression level of EAD is associated with poor overall survival in NPC after radiation therapy. Overall survival rates of NPC patients after radiation therapy with low (n = 46) or high (n = 46) expression levels of EAD were estimated using the Kaplan–Meier method by log-rank test (p < 0.001). **(C)** Cumulative overall survival curves of the combination of 8-OHdG and EAD. NPC patients were classified into 4 groups based on 8-OHdG and EAD expression: Group 1 (n = 31): high 8-OHdG and EAD expression; Group 2 (n = 15): high 8-OHdG but low EAD expression; Group 3 (n = 15): low 8-OHdG but high EAD expression; Group 4 (n = 31): low 8-OHdG and EAD expression (p < 0.001).

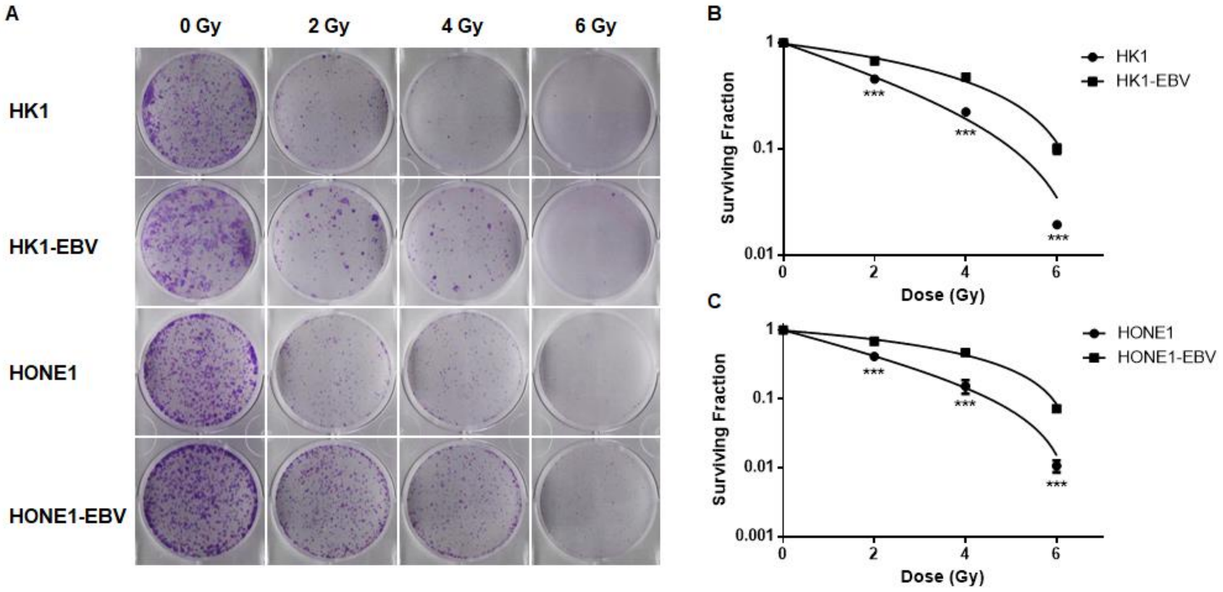

Figure S6. EBV positive cells were resistant to radiation therapy than EBV negative cells. (A) Colony formation of EBV negative and EBV-positive cells with a single dose of 0, 2, 4 or 6 Gy irradiation. **(B-C)** Survival curve of EBV negative and EBV positive cells after a single dose of 0, 2, 4 or 6 Gy irradiation. Surviving fractions were calculated by comparing the colony number of each treatment group with untreated groups (0 Gy). Results are plotted as the mean surviving fraction ± S.D. The survival curves were drawn using the GraphPad Prism 5 software program, ***$p < 0.001$.
